# Cool the Inflamed Brain: A Novel Anti-inflammatory Strategy for the Treatment of Major Depressive Disorder

**DOI:** 10.2174/1570159X21666230809112028

**Published:** 2023-08-09

**Authors:** Wen-Jun Su, Ting Hu, Chun-Lei Jiang

**Affiliations:** 1 Department of Stress Medicine, Faculty of Psychology, Second Military Medical University, Shanghai, 200433, China

**Keywords:** Depressive disorder, cytokines, inflammation, anti-inflammatory drugs, stress, antidepressants

## Abstract

**Background:**

Abundant evidence suggests that inflammatory cytokines contribute to the symptoms of major depressive disorder (MDD) by altering neurotransmission, neuroplasticity, and neuroendocrine processes. Given the unsatisfactory response and remission of monoaminergic antidepressants, anti-inflammatory therapy is proposed as a feasible way to augment the antidepressant effect. Recently, there have been emerging studies investigating the efficiency and efficacy of anti-inflammatory agents in the treatment of MDD and depressive symptoms comorbid with somatic diseases.

**Methods:**

In this narrative review, prospective clinical trials focusing on anti-inflammatory treatment for depression have been comprehensively searched and screened. Based on the included studies, we summarize the rationale for the anti-inflammatory therapy of depression and discuss the utilities and confusions regarding the anti-inflammatory strategy for MDD.

**Results:**

This review included over 45 eligible trials. For ease of discussion, we have grouped them into six categories based on their mechanism of action, and added some other anti-inflammatory modalities, including Chinese herbal medicine and non-drug therapy. Pooled results suggest that anti-inflammatory therapy is effective in improving depressive symptoms, whether used as monotherapy or add-on therapy. However, there remain confusions in the application of anti-inflammatory therapy for MDD.

**Conclusion:**

Based on current clinical evidence, anti-inflammatory therapy is a promisingly effective treatment for depression. This study proposes a novel strategy for clinical diagnosis, disease classification, personalized treatment, and prognostic prediction of depression. Inflammatory biomarkers are recommended to be assessed at the first admission of MDD patients, and anti-inflammatory therapy are recommended to be included in the clinical practice guidelines for diagnosis and treatment. Those patients with high levels of baseline inflammation (*e.g*., CRP > 3 mg/L) may benefit from adjunctive anti-inflammatory therapy.

## 1 INTRODUCTION

Major depressive disorder (MDD) is one of the most prevalent and debilitating diseases in the world. According to estimates by the World Health Organization (WHO), about 280 million people are suffering from this disease, accounting for about 3.8% of the world's total population. The incidence of this disease spreads across all ages, among which the prevalence of depression in adults is about 5%, and the prevalence in people over 60 years old is as high as 5.7% [1]. As MDD often severely limits the psychosocial functioning of patients, reduces their quality of life, and increases economic burden, it is also listed as an important contributor to the global disease burden. In 2018, depressive disorders were reported to be the third leading cause of years lived with disability after low back pain and headache disorders [2, 3]. Especially since the Coronavirus Disease 2019 (COVID-19) pandemic, the incidence of MDD has climbed further. It is reported that the epidemic has increased the prevalence of depression by about 53 million in 2020, an increase of about 27.6%. When we quantified the disease burden using disability-adjusted life years (DALYs), which represent the number of years of healthy life lost due to death or disability, the pandemic was estimated to increase depression-induced DALYs by 137.1/100,000, mainly in females [4]. Therefore, MDD has become an important public health problem that disturbs the global healthcare system, and has attracted worldwide attention.

To date, the treatment of MDD mainly includes three approaches: (1) pharmacotherapy, primarily the application of antidepressants and other adjuvant drugs that enhance the effect of antidepressants; (2) psychotherapy, such as cognitive behavioral therapy (CBT), mindfulness-based cognitive therapy (MBCT) and interpersonal psychotherapy (IPT), and (3) non-drug physical therapy, including electroconvulsive therapy (ECT), transcranial direct current stimulation (tDCS), repetitive transcranial magnetic stimulation (rTMS), and vagus nerve stimulation (VNS) [5-7]. Among them, antidepressant therapy remains the most preferred method, especially for patients with moderate to severe symptoms. In this regard, as early as 1965, Schildkraut *et al*. proposed the “catecholamine hypothesis of affective disorders” based on previous findings that depressive symptoms can be unexpectedly improved by monoamine oxidase inhibitors (MAOIs) or imipramine-like agents [8]. Since then, most of the research and development of antidepressant drugs has focused on maintaining the concentration of the monoamine neurotransmitter and regulating its function. At present, various guidelines for the management of MDD consistently recommend selective serotonin reuptake inhibitors (SSRIs), serotonin noradrenaline reuptake inhibitors (SNRIs), and some other antidepressants as first-line drug options [9]. Generally, most cases of MDD can achieve remission after standardized treatment, while some cases will recur. According to reports, about one-third of patients achieved clinical remission after their first-round antidepressant treatment, and even after 4 different antidepressants were administered sequentially for 12 weeks each time within a year, only two-thirds of patients achieved clinical remission [10, 11]. This leaves a large group of patients with insufficient or no response to currently recommended therapeutic interventions. In addition to the unsatisfactory response and remission, these monoamine-modulating antidepressants also have delayed onset, high recurrence rate, and drug-related side effects, like nausea and sexual dysfunction [9, 12]. Up to 63% of patients taking second-generation antidepressants experience adverse events, while 7% to 15% of patients discontinue treatment due to adverse events [13]. Hence, there is a desire to find an ideal antidepressant with faster action, better toleration, higher efficacy and efficiency, and fewer side effects. Also, there is a strong unmet need for additional treatment options.

To some extent, the appearance of these clinical phenomena may be attributed to the unclear pathophysiological mechanisms of depression. Despite considerable advances in the understanding of the pathophysiology of MDD in recent years, no single model or hypothesis can perfectly explain all aspects of the disorder. On the one hand, different causes or pathophysiological processes may lead to episodes in different patients; on the other hand, even if the same patient has different episodes at different times, the pathogenesis can also be different. Specifically, the most convincing mechanistic hypotheses at present include stress-related disequilibrium, hypothalamic-pituitary-adrenal (HPA) axis dysfunction, monoaminergic neurotransmitter disturbance, pro-inflammatory-anti-inflammatory imbalance, neurotrophic factor deficiency, pathological neurodegeneration, gut microbiota dysbiosis, *etc*. [9, 14-16]. Of these, psychosocial and biological stress seems to be a key origin of MDD [17, 18]. It is associated with almost every mediator underlying the pathophysiology of MDD, including genetic predisposition, history of adverse early life events, HPA axis hyperactivity, decreased monoamines and brain-derived neurotrophic factor, increased proinflammatory cytokines, and even epigenetic alterations [19]. Recently, abundant studies have reported that depressive symptoms and severity are closely related to cytokine levels, and anti-inflammatory drugs are expected to be a promising direction for drug development other than traditional monoamine-regulating antidepressants [20, 21].

In fact, several clinical trials have explored the antidepressant effects of both pharmacological and nonpharmacological therapies aimed at harnessing immune overactivation. However, the methods and results of these studies vary widely: some subjects were inpatients or outpatients diagnosed with MDD, while others were medical patients with depressive symptoms or complaints; some used anti-inflammatory drugs monotherapy, while some applied anti-inflammatory therapy as adjuvants added to treatment as usual; some carried out short-course therapy for a few days, while some performed long-term therapy across years; and tools used to evaluate improvement in depressive symptoms were also substantially different. As such, the main purpose of the current narrative review was to comprehensively assess the literature on the impact of anti-inflammatory treatments on clinical outcomes in patients with MDD, as well as on the effect of these approaches on the severity of depressive symptoms in individuals with somatic disorders. Based on the collected evidence, we have analyzed and discussed the diagnosis and treatment protocols for MDD, and proposed a novel anti-inflammatory strategy, hoping to propose an anti-inflammatory method for diagnosing and treating MDD more reasonably and efficiently.

## 2 INFLAMMATION AND MAJOR DEPRESSIVE DISORDER

As aforementioned, the monoamine hypothesis has dominated the pathogenesis of MDD in the past half-century. The catecholamine [8] and 5-hydroxytryptamine (5-HT or serotonin) [22] hypotheses were proposed almost simultaneously in the 1960s. Although the rationale for these hypotheses arose not from pathophysiological explorations of disease but from unexpected pharmacological observations, it does make a great contribution to the treatment of MDD [23]. However, people have gradually realized that antidepressants designed to modulate monoamine transmitters were unable to meet the practical needs for a complete remission of major depression. Thereupon, human beings try hard to find another way out on this track. Interestingly, the discovery of the linkage between depression and inflammation (cytokines) also arose from unexpected clinical observations [24]. This section will mainly introduce the origin and development of antidepressants, as well as the theoretical background of anti-inflammatory treatment for MDD.

### 2.1 The History and Development of Antidepressants

Antidepressants have been around for hundreds of years. For example, owing to its sweet smell, people smell frankincense to improve the gloomy mood, so it has been used as an “ancient antidepressant” in “aromatherapy” for a period of time [25]. Another botanical candidate is St. John's wort, which was officially listed as an antidepressant in Germany in 1984. In traditional Chinese medicine, it is also known as *Hypericum perforatum* L., and is the main antidepressant medicinal component in Shugan Jieyu Capsule, a proprietary Chinese medicine approved by the State Food and Drug Administration (SFDA) for the treatment of mild to moderate depression [26, 27]. Besides, some psychoactive substances have been suggested as the “solution” to anhedonia. For example, before the formula change in 1904, cocaine was an early ingredient in Coca-Cola, which led to the popularity of this drink in the United States (US) for its effect on inducing excitement and hallucinations. Likewise, benzedrine (amphetamine) and opioids were also marketed in the 1930s and preferred to treat depression [28].

In the 1950s, reserpine was successfully extracted from *Rauwolfia serpentina* (Indian snakeroot) [29]. It was then fully synthesized industrially and brought to the market as an efficient antihypertensive drug [30]. However, doctors soon discovered that reserpine could lead to depression-like symptoms and even severe suicidal ideation. The reason is mainly the mechanism of selectively inhibiting the vesicular monoamine transporter in the intracellular environment, making it difficult for free monoamines (serotonin, norepinephrine, and dopamine) to be transported into the vesicles of the presynaptic nerve terminals in a reuptake manner. As a result, these monoamine neurotransmitters are gradually degraded by the enzymes, monoamine oxidase (MAO) and catechol-O-methyltransferase (COMT), in the cytoplasm [28, 31]. Likewise, iproniazid (isoniazid with a propyl group attached) was also reported to improve mood and appetite possibly by inhibiting MAO and increasing neuronal levels of monoamine [32]. Coincidentally, Swiss psychiatrist Roland Kuhn examined the antidepressant efficacy of the chlorpromazine derivative “imipramine” [33]. Later, in the 1960s, Julius Axelrod, who won a Nobel Prize in 1970, demonstrated that reserpine, amphetamine, imipramine, and chlorpromazine markedly inhibited the reuptake of norepinephrine in cats [34]. Moreover, iproniazid and imipramine were successively approved by the Food and Drug Administration (FDA) for MDD in the US, thus establishing the mechanistic basis of current antidepressant medications.

With the discovery of their antidepressant effects, tricyclic antidepressants (TCAs), such as imipramine and amitriptyline, quickly became the most widely used drugs for MDD, especially those of severe melancholic subtypes. There are also some TCA-associated tetracyclic antidepressants (TeCAs), like amoxapine, maprotiline, mianserin, and mirtazapine [35]. However, most of these drugs and the once popular MAOIs are not well-tolerated due to their adverse impacts, such as anticholinergic and membrane stabilizing (quinidine-like) effects, and the lethality in overdose [36]. Thereby, modern drugs have been developed to be more selective inhibitors of serotonin and norepinephrine reuptake. For example, bupropion was the first member of norepinephrine-dopamine reuptake inhibitors (NDRIs). As the first-line and most prescribed antidepressant category, SSRIs primarily include fluoxetine, paroxetine, sertraline, fluvoxamine, citalopram, and escitalopram. Following the SSRIs, venlafaxine and duloxetine are categorized as SNRIs. Clinical guidelines usually recommend the use of SNRIs for patients who do not respond to SSRIs [37, 38]. Other common antidepressants have also been developed that could both block serotonin reuptake and modulate specific serotonin receptor subtypes [39].

More recently, some antidepressants focusing on other targets have been invented. For instance, mirtazapine exerts its antidepressant effect *via* antagonizing central alpha 2-adrenergic receptors and the 5-HT-2 and 5-HT-3 serotonergic receptors. Because mirtazapine has a limited effect on monoamine reuptake, it was classified as a member of noradrenergic and specific serotonergic antidepressants (NaSSAs) [40]. Agomelatine is a novel antidepressant claimed to act through a combination of agonist activity at MT1/MT2 melatonergic receptors and antagonist activity at 5-HT-2C serotonergic receptors [41, 42]. In 2000, Berman *et al.* [43] first reported that the infusion of N-methyl-D-aspartate (NMDA) receptor antagonist ketamine could rapidly improve depressive symptoms in MDD patients at a relatively low dose within 3 days. Since then, this old anesthetic has expanded into a brand-new field of application. So far, intranasal S-ketamine therapy has been approved by the FDA and the European Medicines Agency (EMA) as a legitimate treatment option for MDD, especially treatment-resistant depression (TRD) [44].

Although an iconic systematic review and network meta-analysis paper that included 21 commonly used antidepressants has concluded that all the candidate drugs were significantly more efficacious than placebo in adults with MDD [23], it is undeniable that their effects are still unsatisfying. As the remarkable sequenced treatment alternatives to relieve depression (STAR*D) study calculated, the cumulative remission rate after four standard steps was still approximately only 67% [5, 11]. Even with optimism, this statistic may not boost much at present. Combined with the delayed clinical onsets and neurochemical adverse effects of most current antidepressants, a purely neurotransmitter-based regimen is challenged.

### 2.2 The Cytokine Hypothesis of Major Depressive Disorder

As also evident in the history, it is very common in medical research that accidental discoveries have provided critical clues. In the late 1980s, McDonald *et al.* [45] carried out a clinical trial administering interferon-α (IFN-α) to 43 hepatitis B patients for 3-6 months. This far-reaching study showed that participants who received IFN-α had a significant increase in psychiatric symptoms, some severe enough to require urgent psychiatric treatment, compared to a matched control group. These patients often complained of fatigue, loss of interest, poor concentration, low mood, and anxiety. Subsequently, a smaller but more purposeful clinical study reported similar emotional changes in IFN-treated patients. Niiranen *et al.* [46] assessed behavioral and subjectively experienced changes in 9 patients with small cell lung cancer (SCLC) treated with IFN-α infusions. Although the recruited patients were confirmed mentally healthy before the trial, after 6 days of IFN-α treatment, all patients complained of depression-like symptoms, including moderate to severe fatigue, lack of interest, slowed thinking, loss of appetite, anorexia, confusion, or inability to concentrate. Besides, 7 of the 9 became irritable, and 5 experienced severe depressed mood. By integrating these clinical clues, the cytokine hypothesis of depression was first proposed by Smith [20] in the form of the “macrophage theory of depression,” and further demonstrated and elucidated by Maes *et al.* [47] in the early 1990s [24, 48].

Cytokines are soluble proteins with a wide range of biological activities. They are mainly secreted by immune cells, like monocytes, macrophages, lymphocytes, microglia, and astrocytes, as well as by non-immune cells, like endothelium, epidermis, and fibroblasts [49]. In short, they play a pivotal double-edged role in maintaining innate and acquired immune defense responses against pathogens and tumors, as well as regulating the homeostasis of harm and self-repair. According to the different biological functions of inflammation-related cytokines, they can be roughly divided into two categories: pro-inflammatory cytokines and anti-inflammatory cytokines. As the name suggests, they are involved in promoting or restricting inflammation, respectively [50]. For example, interleukin-1β (IL-1β), interleukin-6 (IL-6), tumor necrosis factor-α (TNF-α), and IFNs are some of the well-studied pro-inflammatory cytokines (also known as inflammatory cytokines or inflammatory factors), while interleukin-4 (IL-4) and interleukin-10 (IL-10) are common anti-inflammatory cytokines [51].

In the past 30 years since the cytokine hypothesis of depression has been proposed, mounting evidence has confirmed the pathophysiological mechanisms of depression to be closely associated with the inflammatory imbalance and cytokine secretion. Conclusively, the association between inflammation and major depression mainly includes the following aspects [52]: (1) Patients with MDD often have abnormal levels of pro-inflammatory or anti-inflammatory cytokines [53, 54]. In 2010, a meta-analysis recruited 24 studies involving unstimulated measurements of cytokines in patients meeting the Diagnostic and Statistical Manual of Mental Disorders-4 (DSM-4) for MDD and reported significantly higher levels of the proinflammatory TNF-α and IL-6 in depressed individuals than matched controls [55]. Similarly, more recent meta-analyses have also concluded that, in addition to TNF-α and IL-6, peripheral levels of IL-18, IL-10, the IL-1 receptor antagonist, the soluble IL-2 receptor, C-C chemokine ligand 2, IL-13, IL-12, and the soluble TNF receptor 2 [56], as well as IL-6 and TNF-alpha levels in CSF and brain parenchyma [57] are elevated in patients with MDD compared to healthy controls. (2) Inflammation-related diseases, including rheumatoid arthritis [58], inflammatory bowel disease [59], psoriasis [60], systemic lupus erythematosus [61], coronary artery disease [62], and cancer [63], are often co-morbid with depression [64]. (3) Administration of exogenous pro-inflammatory cytokines, like IFN-α [65], or experimental endotoxemia induced by lipopolysaccharide injection [66], result in sickness behavior or mood impairment in humans. (4) Antidepressants and other effective treatments for MDD can reduce the level of inflammatory cytokines in patients with major depression to a certain extent [67]. Several drugs that modulate serotonergic signaling, including SSRIs and SNRIs, have also been shown to affect peripheral immunity [68-70]. Furthermore, depressive patients unresponsive to SSRIs have been reported to have higher peripheral IL-6 and TNF-α concentrations compared to normal controls and patients previously resistant to SSRIs [71]. Likewise, a poor outcome following psychological therapy is associated with elevated IL-6, soluble intracellular adhesion molecule-1 (sICAM1), and TNF-α levels, and attenuated IFN-γ level [72]. (5) The level of peripheral inflammatory factors is related to the severity and prognosis of depression [73-75]. Moreover, inflammation has been reported to be highly associated with suicidal ideation and suicidal attempts, both in depressed and non-depressed populations [76-78]. It has also been speculated that cytokines take part in the biomechanism of suicide possibly *via* interfering with monoamine metabolism and related pathways [79].

### 2.3 Stress, Inflammation, and Neuroinflammation

As illustrated above, most clinical evidence for the relationship between major depression and inflammatory cytokines (suggestive of chronic low-grade inflammation) has been obtained in peripheral samples. However, in the traditional view, major depression is more of a disorder related to the dysfunction of the central nervous system (CNS) and less of an infection-associated disorder. That is to say, the “real” facilitator of MDD may be a sterile systemic inflammatory response [80]. So the questions are, where does this particular type of inflammation come from, and how do central and peripheral cytokines interact?

In general, the peripheral and central innate immune system can be activated by signaling molecules derived from exogenous or endogenous factors, which are also known as pathogen-associated molecular patterns (PAMPs) or danger-associated molecular patterns (DAMPs), respectively [81]. Although pattern recognition receptors (PRRs) on the surface of immune cells can recognize both PAMPs and DAMPs, the ways in which they elicit immune responses vary widely: (1) PAMP-PRR signaling typically functions upon exposure to foreign microbes or viruses, whereas (2) innate immune activation triggered by DAMP-PRR signaling usually occurs in the absence of exogenous pathogens or other sources of infection [80]. Unfortunately, the type, release, and regulatory mechanism of these DAMPs that trigger sterile inflammation have not been fully elucidated. Nevertheless, it is suggested that their release is mostly related to neuroendocrine and homeostatic changes induced by physical and psychological stress, which is one of the most important causes of MDD [80].

Under stressful situations, the HPA axis is activated, which subsequently stimulates the adrenal glands to synthesize and secrete cortisol into the blood, thereby leading to a systemic increase in peripheral and central cortisol levels. When the glucocorticoid receptor (GR) in the hippocampus detects elevated cortisol levels, it modulates the hypothalamus through negative feedback to lower corticotropin-releasing hormone (CRH) [82]. Although still controversial, emerging evidence supports elevated peripheral cortisol levels and reduced GR sensitivity in many subgroups of patients with MDD [82-85]. HPA axis dysregulation seems to be a vulnerability factor for MDD [86]. In addition, stress-induced activation of the sympathetic nervous system acts systematically in multiple organs, causing neuroendocrine and immune responses [18, 87]. In humans, there is clinical evidence that various types of psychosocial stressors may disrupt the balance of cytokine networks, including increasing pro-inflammatory IL-6, TNF-α, and IFN levels, and decreasing anti-inflammatory IL-10 level [88-90]. Given the complex effects of stress on the human body, experimental animals are widely used to mimic stress responses for further investigation. Studies have shown that different types of stressors could increase the pro-inflammatory cytokines (including IL-1, IL-6, and TNF-α) in the circulation and CNS, and enhance the production and secretion of DAMPs, like high mobility group box 1 protein (HMGB1), S100b proteins, heat shock proteins (HSP), *etc*. [80, 91] Typical pathophysiological mechanisms may include activating transcriptional factors, like nuclear factor-κB (NF-κB), enhancing cyclooxygenase-2 (COX-2) and prostaglandin production, and facilitating cell death (apoptosis/necroptosis/pyroptosis) [92-94].

The brain was once considered to be protected from pathogens and immune cells because of the blood-brain barrier (BBB). This feature of “immune privilege” was originally understood to be the result of the organ's lack of any lymphatic drainage [95], which has now been revised. To the best of our knowledge, there are two main pathways for the origin and development of neuroinflammation. First, it may be associated with peripheral inflammation. Although cytokines are relatively large proteins, they can cross the BBB in a leaking way through circumventricular organs [96]. Stress and the increased C-reaction proteins (CRPs) can also enhance the permeability of the BBB, allowing further entry of cytokines and CRPs into the brain [97]. Second, another penetration mechanism has also been demonstrated, namely through specific saturable BBB transporters [98, 99]. Besides, cytokine signaling can be transmitted to the brain by activating cytokine receptors on afferent nerve fibers [100]. Also, the endothelial cells of the BBB can secrete cytokines [101]. In addition to its peripheral origin, the CNS also has its own “local immune system”. Microglia, which share common progenitor cells with peripheral macrophages, are resident immune cells in the brain that produce pro-inflammatory cytokines, nitric oxides, and reactive oxygen species [102]. Moreover, when microglia are activated in response to increased peripheral inflammation, they can produce chemokines that attract monocytes to the brain [103], and further produce pro-inflammatory cytokines [104]. On one hand, the increase in pro-inflammatory cytokines leads to GR resistance by disturbing GR function, causing the hyperactivation of the HPA axis [49], while on the other hand, the increased pro-inflammatory cytokines may have implications for brain structure and function, possibly by increasing neurotoxic metabolites through the kynurenine pathway or by exerting neurotoxic effects directly on specific brain regions. Recently, a meta-analysis review of positron emission tomography (PET) neuroimaging studies examining the 18-kDa translocator protein (TSPO) suggested the presence of TSPO upregulation in MDD, especially in the anterior cingulate cortex (ACC), prefrontal cortex (PFC), hippocampal formation and insula [105]. This result indicates a change in the phenotype and functional state of microglia in patients with MDD.

## 3 ANTI-INFLAMMATORY TREATMENT FOR MAJOR DEPRESSIVE DISORDER

In order to retrieve research data for prospective clinical trials of anti-inflammatory treatments for MDD, we searched PubMed for studies published from 1966/01 to 2022/08/31. Briefly, the search consisted of two main components: (1) anti-inflammatory treatments and (2) depression. By referring to previous reviews, “anti-inflammatory treatments” was searched as the following medical subject heading when possible or free text words with slight modifications [106]: anti-inflammatory, non-steroidal anti-inflammatory, NSAID, cyclooxygenase 2 inhibitor, COX-2, celecoxib, acetaminophen, acetylsalicylic acid, cytokine inhibitors, antibodies monoclonal, infliximab, etanercept, antibiotics, minocycline, statin, corticosteroid, glucocorticoid, peroxisome proliferators-activated receptors agonist, PPAR agonist, immunomodulator, or immunosuppressor. Simultaneously, “depression” was searched as major depressive disorder, depression, or depressive symptoms. The search results of the two parts were combined using the “AND” operator, and then automatically screened by applying the filter function for the clinical trial. It should be noted that the current paper is a comprehensive narrative review in which the included studies are reorganized by drug types, rather than a systematic review exhaustively reviewing the literature or a meta-analysis integrating results and quantifying the effect size. Data were impartially compiled and overviewed as systematically as possible. The results are described below. A summary of the original studies included in this review can be found in Table **1**.

### 3.1 Cytokine Inhibitors

As a canonical pro-inflammatory cytokine, TNF-α plays a protective role against infection and injury in normal immune responses. Upon binding to TNF receptors (TNFR1/ TNFR2), TNF-α mediates apoptosis and inflammatory cascades by activating NF-κB, mitogen-activated protein kinase (MAPK) pathways, and death signaling. There are three common types of TNF-α antibodies approved for clinical use: (1) anti-TNF-α IgG1 antibodies (infliximab/Remicade, adalimumab/Humira, and golimumab/Simponi), (2) pegylated antibody fragment (certolizumab pegol/Cimzia), and (3) antibody-like Fc-fusion protein (etanercept/Enbrel) [107].

Adalimumab is the first fully recombinant human subcutaneously administered anti-TNF monoclonal (mAb) antibody that is structurally and functionally indistinguishable from naturally occurring human immunoglobulin 1 (IgG1) [108]. It specifically binds to TNF-α and blocks interactions with p55 and p75 cell surface TNFRs [109]. The study by Loftus *et al.* [110] included patients diagnosed with moderate to severe Crohn’s disease for at least 4 months prior to the beginning of the study. At the baseline visit (week 0), all patients received open-label adalimumab 80-mg subcutaneous injection, followed by a 40-mg dose at week 2 (induction therapy). At week 4, a total of 778 patients identified as “responders” were randomized to three groups: adalimumab 40 mg every other week (eow), adalimumab 40 mg weekly, or placebo injections (“induction-only” group). In addition to other health-related quality of life (HRQOL) measurements, 499 randomized responders (with a reduction of at least 70 points from baseline in the Crohn’s Disease Activity Index [CDAI]) were evaluated and analyzed with the Zung Depression Scale (ZDS) at baseline and weeks 4, 12, 26, and 56. Compared to the placebo group, patients in the adalimumab EOW maintenance group reported less ZDS scores at all time blocks after week 4, while patients who continued weekly injections exhibited less depression only at week 56. There were no significant differences in ZDS scores between the adalimumab EOW and weekly arms for all visits. However, a possible bias could be that the analyses were primarily based on “responders” to induction therapy. Menter *et al.* [111] investigated the effects of adalimumab on depression symptoms in anti-TNF therapy-naïve patients with moderate to severe psoriasis (body surface area ≥ 5%) that could not be controlled by topical therapy. A total of 96 included patients were randomly assigned to double-blind treatment with placebo or adalimumab 40 mg weekly, or EOW treatment starting at week 1 after the 80 mg dose at baseline. But, in this study, the authors only analyzed the ZDS scores in the 40-mg EOW and placebo groups at baseline and week 12 or early termination (ET). Compared to the placebo group, the ZDS score in the EOW group reduced more by week 12/ET.

Infliximab is the first chimeric mAb (mouse/human) designed to block and neutralize TNF-α [112]. Although not the first to try, Raison and collaborators [113] published the first paper to examine TNF-α-inhibitory therapy for MDD in antidepressant nonresponders. In this trial, 60 MDD patients who were either receiving a consistent antidepressant regimen or medication free for 4 weeks were included and randomized to a treatment group with 3 infusions of 5 mg/kg infliximab or placebo. Group assignment was stratified by sex and baseline inflammation as determined by high-sensitive CRP (hs-CRP) levels. Assessments of the 17-item Hamilton Scale for Depression (HDRS) and inflammatory status (hs-CRP and TNF, as well as its soluble receptors I and II) were conducted at baseline and weeks 1, 2, 4, 6, 8, 10, and 12. Like previous traditional trials for antidepressants, the primary outcome of this study was a change in HDRS scores, while additional outcomes included treatment response (50% or more reduction in HDRS score at any time) and remission (HDRS score ≤ 7 at the end of treatment). At first glance, infliximab did not show overall superiority to placebo on the primary outcome. However, the subsequent analysis demonstrated a significant interaction between treatment, time, and log baseline hs-CRP level. For patients with higher baseline inflammation (hs-CRP > 5 mg/L), treatment with infliximab resulted in greater HDRS reduction and better response (not statistically significant) than placebo, while placebo outperformed infliximab in patients with lower baseline inflammation (hs-CRP ≤ 5 mg/L). It should be noted that this study also included participants with bipolar disorder and depression. The second analysis of this trial also showed that compared to infliximab-treated patients with hs-CRP ≤ 5 mg/L, sleep continuity of those with hs-CRP > 5 mg/L significantly improved from baseline to 2 weeks after 3 infusions (week 8) [114]. Furthermore, a recent study by Webers *et al.* [115] evaluated the effect of infliximab on depressive symptoms in adult patients diagnosed with ankylosing spondylitis (AS). The recruited patients were randomly allocated to infliximab or placebo until week 24 (infusions of 5 mg/kg infliximab or placebo at weeks 0, 2, 6, 12, and 18), after which all received infliximab until week 54. Depressive symptoms were measured with the Center for Epidemiological Studies Depression scale (CES-D). Finally, the results showed that after 24 weeks of treatment, the infliximab group had a greater improvement in CES-D scores than the placebo group. However, the small number of patients and the application of a screening questionnaire somewhat limited the power of this study.

Etanercept is a dimeric fusion protein that binds TNF, which competitively inhibits the binding of TNF to TNFRs on the cell surface, rendering TNF biologically inactive [116]. Kekow and colleagues [117] conducted a trial to compare the effect of etanercept 50 mg once weekly combined with methotrexate (MTX) *versus* MTX alone on patient-reported outcomes (PROs), including Hospital Anxiety and Depression scale (HADS), for 52 weeks. Although most PROs exhibited significantly greater improvements with etanercept plus MTX than MTX alone, the result regarding HADS was negative. Another study by Tyring *et al.* [118] evaluated the effect of etanercept in patients diagnosed with moderate-to-severe plaque psoriasis with scalp involvement. The patients were randomly allocated to two groups. One group received etanercept 50 mg twice weekly (BIW) by subcutaneous injection for 12 weeks, followed by etanercept 50 mg once weekly (QW) and placebo QW for 12 weeks. The other group received placebo BIW for 12 weeks, followed by etanercept 50 mg BIW for 12 weeks. A meaningful difference in depressive symptoms, as assessed by PROs of emotional distress/depression, between etanercept and placebo, was observed at week 12.

As introduced before, another cytokine that is strongly involved in depression is IL-6. Accordingly, Sun *et al.* [119] performed a study to examine the effect of IL-6 mAbs administration on depressive symptoms in patients with multicentric Castleman’s disease (MCD) or rheumatoid arthritis (RA). The data were obtained from two independent trials to test the efficacy of siltuximab on MCD and sirukumab on RA. Siltuximab is a chimeric anti-IL-6 antibody with murine variable and human constant regions, which inhibits the binding of IL-6 to IL-6 receptor, while sirukumab is a fully human anti-IL-6 mAb that binds to a special site of IL-6 [120]. Results showed that both siltuximab treatment for 15 weeks and sirukumab treatment for 12 weeks significantly improved depressive symptoms compared to placebo, as assessed by the 36-item Short Health Questionnaire (SF-36).

Considering that IL-6 plays a specific role in enhancing inflammation by regulating T helper cell 17 (Th17) and IL-17 production, and that the IL-17A pathway may have potential CNS effects, Griffiths *et al.* [121] conducted a clinical trial to determine the efficacy of anti-IL-17A mAb in treating depressive symptoms in patients with moderate-to-severe psoriasis. The data were integrated from 3 phase III trials. Patients were randomly assigned to receive 80 mg ixekizumab (a humanized high-affinity mAb that selectively inhibits the binding of IL-17A to IL-17 receptor A) every 4 weeks (q4w), and 80 mg ixekizumab or placebo every 2 weeks (q2w). Serum hs-CRP and 16-item Quick Inventory of Depressive Symptomology - Self-Report (QIDS-SR16) were assessed at baseline and week 12. Pooled data from 2 of the 3 studies were also used for the comparison of etanercept. At week 12, patients treated with both doses of ixekizumab showed significantly greater reductions in their QIDS-SR16 total scores and higher rates of remission of depressive symptoms *versus* placebo. Moreover, patients treated with ixekizumab also had significant reductions in hsCRP. However, there was no significant difference found between the etanercept treatment group and the placebo group in terms of their changes in QIDS-SR16 total score or clinical response and remission.

As reported, IL-23 is a pro-inflammatory cytokine that promotes the development of Th17 cells. Ustekinumab is a human mAb targeting the p40 subunit found in both IL-12 and IL-23 [122]. An open-label trial validating the effect of ustekinumab on depressive symptoms in patients with moderate to severe psoriasis was performed by Kim and collaborators [123]. In this study, 15 sex-matched healthy volunteers were recruited for comparison. Treatment with 45 mg of ustekinumab (3 injections at baseline, weeks 4 and 12; 90 mg if baseline weight ≥ 100 kg) significantly reduced depressive symptoms, as evaluated using Beck Depression Inventory (BDI) and HDRS. However, the brain 18-fluorodeoxy-glucose (FDG)-PET detection showed no significant difference between the groups.

Additionally, the effect of dupilumab, a fully human mAb targeting the IL-4 receptor-α and inhibiting signaling of the Th2 cytokines IL-4 and IL-13, on PROs, including depressive symptoms in adults with moderate to severe atopic dermatitis, was assessed by Simpson *et al.* [124]. Patients were randomly allocated to subcutaneous treatment with dupilumab 100 mg every 4 weeks (q4w), 300 mg q4w, 200 mg q2w, 300 mg q2w, 300 mg qw, or placebo for 16 weeks. At the 16th week, patients receiving all doses of dupilumab reported greater reductions in total HADS and depression subscale scores. Further, Cork *et al.* [125] showed that dupilumab at 300 mg q2w and 300 mg qw doses significantly improved the depressive symptoms of patients with atopic dermatitis after 2 weeks of treatment, which lasted until the whole 16-week treatment phase, compared to placebo.

### 3.2 NSAIDs

Nonsteroidal anti-inflammatory drugs (NSAIDs) are one of the most commonly used drugs for the treatment of pain, fever, and inflammation [126]. Their basic mode of action is to inhibit cyclooxygenase (COX) enzymes, thus reducing the release of prostaglandins (PGs) and thromboxane (TxA) [127]. Generally, COX-1 is constitutively expressed at low levels in organs, such as the gastrointestinal tract and kidney, and maintains the synthesis of physiologically important prostanoids. In contrast, the expression of COX-2 is up-regulated in immune cells by pro-inflammatory stimulants. Sustained activation of COX-2 is associated with the overproduction of inflammatory factors/mediators and pain-related PGs [127, 128]. The classification of NSAIDs drugs is also usually based on chemical structure and COX selectivity: (1) non-selective inhibitors include acetylated salicylates (aspirin), (2) nonacetylated salicylates (diflunisal), (3) acetic acid (indomethacin, diclofenac), (4) propionic acid (ibuprofen, naproxen), (5) enolic acid (piroxicam, meloxicam), and (6) more selective COX-2 inhibitors (celecoxib, etoricoxib) [129]. Those with highly selective inhibition of COX-2 are thought to have fewer side effects and are preferable because they not only prevent the release of excessive pro-inflammatory PGE2, but also spare the gastroprotective prostaglandin synthesis mediated by constitutive COX [127].

There are many clinical trials using COX-2 selective inhibitors to treat depression. According to a clinical trial conducted by Muller *et al.* [130], in patients suffering from an acute depressive episode, 6 weeks of combined treatment with celecoxib (400 mg/day) and reboxetine resulted in a more significant improvement in HDRS scores compared to reboxetine plus placebo. Another study by Akhondzadeh *et al.* [131] included 40 adult outpatients diagnosed with MDD by DSM-IV criteria (HDRS score ≥ 18). The celecoxib group received fluoxetine 40 mg/day plus celecoxib 400 mg/day (200 mg bid) while the placebo group received matched fluoxetine plus placebo. Evaluations were performed using HDRS at baseline and 1, 2, 4, and 6 weeks after the active phase of the study. Although HDRS scores in both groups decreased over the trial period, the combination of fluoxetine and celecoxib showed a significant superiority over fluoxetine plus placebo. Similarly, Abbasi *et al.* [132] showed that the combination of 200 mg twice daily celecoxib with 200 mg/day sertraline for 6 weeks yielded a significantly greater reduction in HDRS scores and serum levels of IL-6 compared to placebo. Jafari *et al.* [133] investigated the use of celecoxib in individuals with mild-to-moderate depression due to brucellosis and scored less than 19 on HDRS-17. The patients were separated into groups of celecoxib (200 mg bid) or placebo as an adjunctive to antibiotic therapy (600-900 mg/day rifampin added to 100 mg doxycycline twice daily) for 8 weeks. Depressive symptoms were evaluated using HDRS at baseline, week 4, and week 8. The results showed the celecoxib-combination group to have significantly lower HDRS scores since week 4, as well as a greater reduction in HDRS and a higher response from baseline to week 8 compared to the placebo-combination group. However, since the use of the antibiotic itself may alter the immune system, this study may be biased. The study by Majd *et al.* [134] included female patients diagnosed with MDD of the first episode by DSM-IV-TR (text revision) criteria. The patients were treated with either sertraline plus celecoxib (100 mg bid) or sertraline plus placebo twice daily, and evaluated by HDRS at baseline, week 4, and week 8 of treatment. Although the celecoxib group had a greater reduction in HDRS scores and a higher response after 4 weeks compared to the placebo group, these effects did not last until the 8th week. The results suggested that celecoxib may hasten the onset of the antidepressant action of sertraline. Furthermore, Alamdarsaravi and collaborators [135] conducted a trial to evaluate the safety and efficacy of celecoxib monotherapy (200 mg bid) for depressive symptoms in colorectal cancer patients undergoing chemotherapy. The recruited participants had mild to moderate depression according to the DSM-V, with an HDRS score ranging from 8 to 18. Regarding the result, improvements in HDRS scores were significantly greater in the celecoxib group by week 2 and remained higher by week 6 post-intervention. Besides, although there was no significant difference in response rates between the two groups, the celecoxib group showed a higher response rate at week 6. In another recent study, Baune *et al.* [136] performed a clinical trial to measure the efficacy of anti-inflammatory augmentation of antidepressant treatment in MDD patients diagnosed by Mini-International Neuropsychiatric Interview (MINI) and DSM-IV. The study was designed similarly to previous trials; participants with a Montgomery-Asberg Depression Rating Scale (MADRS) score ≥ 20 were assigned to receive vortioxetine plus celecoxib (400 mg daily) or vortioxetine plus placebo for 6 weeks. The authors obtained negative results that celecoxib augmentation did not show superior efficacy over placebo on depressive symptom severity, response and remission rates, cognition and psychosocial functioning. There was also no evidence that pretreatment inflammation levels were associated with the effect of the celecoxib co-administration compared to placebo.

In a study aimed to determine the effect of low-dose aspirin (100 mg daily) on the risk of depression in healthy elderly adults (≥ 70 years), the results failed to support the primary hypothesis that individuals taking low-dose aspirin would have a lower prevalence of depression compared to those taking placebo. This study defined depression as a score of at least 8 on the 10-item CES-D (CES-D-10) scale [137]. Another study by Berk *et al.* [138] explored the antidepressant effects of NSAIDs and rosuvastatin in addition to treatment as usual in young people (aged 15-25 years) with moderate to severe MDD (MADRS score ≥ 20). Participants were assigned to receive either aspirin (100 mg daily), rosuvastatin (10 mg daily), or placebo in addition to treatment as usual. There were no significant differences found in the improvement of depression between rosuvastatin and placebo, or between aspirin and placebo. In patients aged ≤ 18 years, MADRS scores reduced more in the rosuvastatin group at week 8, and in the aspirin group at weeks 4 and 8, compared to the placebo group. Further, statins were superior to aspirin on the MADRS at week 12. Additionally, Mahagna *et al.* [139] carried out a study to investigate the efficacy and safety of adding etoricoxib *vs.* placebo to the current therapeutic regimen of female patients with fibromyalgia. Patients were randomized to receive 90 mg once-daily etoricoxib or matched placebo for 6 weeks and were evaluated for depression by HDRS. Although being mildly underpowered, this study clearly showed that etoricoxib conferred no beneficial effects on HDRS in female patients with FMS. But owing to the adverse effects on the cardiovascular system, etoricoxib is not approved for use in several countries [127].

In addition to the abovementioned clinical trials comparing NSAIDs to placebo, several studies have compared the antidepressant effects of different types of NSAIDs. Field *et al.* [140] performed a study to assess the effect of celecoxib and naproxen on depressive symptoms in older adults. Cognitively normal participants aged at least 70 years with a family history of Alzheimer-like dementia were randomly assigned to treatment with celecoxib 200 mg bid, naproxen sodium 220 mg bid, or placebo. The 30-item version of the Geriatric Depression Scale (GDS) was used to measure depressive symptoms at enrollment and at yearly follow-up visits, while participants with a GDS score > 5 at baseline were identified as depressed. The results of this study were negative, showing that neither celecoxib nor naproxen improved depressive symptoms over time compared to the placebo. Iyengar and collaborators [141] conducted a study to investigate the possible antidepressant effect of three NSAIDs in patients with active osteoarthritis. This study pooled data from 5 postapproval trials. Patients were randomly distributed to receiving ibuprofen 800 mg 3 times daily or naproxen 500 mg twice daily, celebrex 200 mg daily or placebo for 6 weeks. Depression was evaluated using the Patient Health Questionnaire-9 (PHQ-9). The results showed that the ibuprofen or naproxen group and the celebrex group exhibited a detectable effect in reducing the PHQ-9 score. Regarding changes in depression classification, logistic regression analysis showed a trend towards a significant treatment effect for all the included NSAIDs compared to placebo. Another study was performed by Mohammadinejad and colleagues [142] to compare the efficacy and safety of selective inhibitors of COX-2 with nonselective inhibitors of both COX-1 and COX-2 in reducing depressive symptoms of patients with breast cancer. Fifty-two outpatients aged 18-70 years with breast cancer and mild to moderate depression (HDRS score ≤ 18), who suffered from pain and needed analgesics, were randomly divided to receive treatment with celecoxib (200 mg bid) or diclofenac (50 mg bid) for 6 weeks. The results showed that in the 6th week, the HDRS score of the celecoxib group was significantly improved compared to that of the diclofenac group.

### 3.3 Antibiotics

Although the intended activity of antibiotics is to kill or prevent the growth of harmful pathogenic microorganisms, these drugs may also directly affect host factors unrelated to the infection. Some of these antibiotics are neuroprotective, such as minocycline, a synthetic broad-spectrum antibacterial tetracycline that exerts neuroprotective function through anti-apoptotic and anti-inflammatory mechanisms [143], and rapamycin, an antifungal agent with apoptotic and pro-autophagic properties that provide neuroprotective effect [144].

To a certain extent, the ability of minocycline to prevent microglial activation may overlap with its anti-inflammatory properties. It could efficiently cross the BBB and inhibit microglial proliferation by interfering with p38 MAPK, thereby reducing cytokine release and cytokine receptor expression [145, 146]. Emadi-Kouchak *et al.* [147] investigated the antidepressant effects of minocycline in human immunodeficiency virus (HIV) infected patients with mild-to-moderate depression (HDRS score up to 18) in a trial. In addition to highly active antiretroviral therapy (HAART), the included patients were randomized to receive minocycline (100 mg bid) or a matching placebo for 6 weeks. Results showed a significant improvement in HDRS scores from baseline to weeks 3 and 6 in the minocycline group compared to the placebo group. Besides, there were significantly more partial responders (as defined by a 25-50% reduction in HDRS score) in the minocycline group than in the placebo group at both weeks 3 and 6. However, the study conducted by Dean *et al.* [148] examined adjunctive minocycline in addition to treatment as usual for MDD. Patients diagnosed with MDD by DSM-IV criteria (MINI PLUS 5, MADRS score ≥ 25) were allocated to treatment with 200 mg/day of minocycline or placebo for 12 weeks. An extra follow-up was also conducted 4 weeks after the end of the treatment phase. There were no significant differences observed between minocycline and placebo groups in MADRS score, MADRS reduction, response rate, and remission rate during 12 weeks of treatment and 4 weeks after treatment (week 16). Similarly, Husain and collaborators [149] performed a smaller-sample study where the inclusion criteria were a diagnosis of TRD based on the DSM-V criteria. In addition to treatment as usual, the individuals also received minocycline (100 mg once daily and increased to 200 mg after 2 weeks) or a matching placebo for 12 weeks. The researchers obtained positive results with a significant reduction in HAMD scores in the minocycline group compared to the placebo group. It is important to note that most of the participants in this trial were receiving SSRI antidepressants as part of their usual care, and most were also using an augmenting agent. Recently, Nettis *et al.* [150] conducted a 4-week clinical trial of 39 patients with elevated serum CRP levels (hs-CRP ≥ 1 mg/L) to test whether minocycline enhanced antidepressant efficacy in patients with TRD (HDRS score ≥ 14). The patients were randomized to receive minocycline (200 mg/day) or a placebo added to treatment as usual. The HDRS was used to assess symptom severity and treatment response. Interestingly, in the specially selected sample of patients with elevated CRP (≥ 1 mg/L), minocycline and placebo did not differ significantly in the improvement of depressive symptoms at week 4. However, patients with baseline hs-CRP levels ≥ 3 mg/L had a substantially increased response to minocycline compared to the other groups. In another trial, Attwells *et al.* [151] demonstrated no significant differences between minocycline and placebo groups in reducing HDRS, and in altering TSPO VT within PFC, ACC, and insula in patients with TRD. These results were obtained through HDRS assessment and [18F]FEPPA PET scan before and after oral administration of minocycline 100 mg bid or placebo in a randomized double-blinded manner for 8 weeks.

In terms of rapamycin, it was first identified in a *Streptomyces* isolate found on Easter Island and later developed as an antifungal agent [152]. Recently, the antifungal ability of rapamycin has gradually been supplanted by its immunosuppressive and anticytoproliferative properties. It is now approved and widely used to prevent organ transplant rejection and cancer treatment [145]. Meanwhile, ketamine is an N-methyl-D-aspartate receptor (NMDAR) antagonist that exerts a rapid and powerful antidepressant effect. The mechanism by which this drug and its metabolites exert their antidepressant effects is thought to be primarily related to the activation of synaptic α-amino-3-hydroxy-5-methyl-4-isoxazolepropionic acid glutamate receptors (AMPARs) by inducing a surge in prefrontal glutamate neurotransmission. Subsequently, the increased brain-derived neurotrophic factor (BDNF) activates the TrkB receptor, which in turn activates the mechanistic target of rapamycin complex 1 (mTORC1), thus leading to synaptogenesis [153-156]. In line with this, Abdallah *et al.* [157] tested the effect of rapamycin on the antidepressant effect of ketamine. In the double-blind crossover-designed clinical trial, 20 patients with MDD were randomly assigned to receive pretreatment with oral rapamycin (6 mg) or placebo 2 hours before the open-label intravenous infusion of ketamine (0.5 mg/kg). The time between the separated crossover treatments was at least 2 weeks apart, and depression severity was evaluated by MADRS. Rapamycin pretreatment did not alter the antidepressant effect of ketamine in terms of HDRS scores at 24 hours post-injection. But over the subsequent 2 weeks, the authors found a significant treatment interaction with time, showing that rapamycin prolonged the antidepressant effects of ketamine. Two weeks after the ketamine infusion, response and remission rates were higher with rapamycin plus ketamine than with placebo plus ketamine.

### 3.4 Antidiabetic Drugs with Anti-inflammatory Properties

In recent years, many studies have reported that depression and diabetes are highly comorbid and influence each other. Considering the intrinsic link between them, and the potential anti-inflammatory and neuroprotective effects of antidiabetic drugs [158], the possible effects of antidiabetic drugs on depressive symptoms have been examined. Technically, the most used antidiabetic drug classes include biguanides, sulfonylureas, thiazolidinediones (TZDs), α-glucosidase inhibitors (AGIs), dipeptidyl peptidase-4 inhibitors (DPP-4i), glucagon-like peptide 1 receptor agonist (GLP-1RA), and sodium-glucose cotransporter type 2 inhibitor (SGLT2i) [158].

Pioglitazone, a TZD antidiabetic drug, is a highly selective peroxisome proliferator activator gamma receptor (PPAR-γ) agonist. Its main mechanism of action is to activate PPAR-γ, thereby sensitizing the transcription of insulin-responsive genes and regulating the production, transportation, and utilization of glucose. In addition, PPAR-γ possesses important anti-inflammatory, neuroprotective, antioxidant, and anti-excitotoxic properties [159]. The first double-blind placebo-controlled study addressing the efficacy of pioglitazone in MDD was performed by Sepanjnia *et al.* [160]. The inclusion criteria were a diagnosis of MDD according to the DSM-IV-TR criteria and an HDRS score ≥ 22. After the screening, the patients were separated into groups that received citalopram plus pioglitazone (15 mg every 12 h) or matching citalopram plus placebo for 6 weeks. HDRS was used to evaluate depressive symptoms at baseline and at weeks 2, 4 and 6. The results showed that HDRS scores in the pioglitazone group were significantly lower than those in the placebo group throughout the treatment period. Also, the response and remission rate were significantly higher in the pioglitazone group compared to the placebo group. Subsequently, Lin *et al.* [161] conducted a study to evaluate the effects of adjuvant therapy with the PPAR-γ agonist pioglitazone on depressive symptoms. Patients with non-psychotic, non-remitting depression receiving treatment as usual for depression were randomized across an insulin sensitivity spectrum to the treatment as usual plus pioglitazone (30 mg/day) or a matching placebo for 12 weeks. Clinical tests, including glucose tests (baseline and week 12) and HDRS (baseline and weeks 2, 4, 6, 8 and 12), were used to assess systematic improvements. Although there was a significant decrease in HDRS scores in both pioglitazone and control groups from baseline to week 12, the decline in HDRS scores did not differ significantly between the two groups. Besides, in the pioglitazone group, changes in HDRS were positively correlated to changes in the oral glucose tolerance test (OGTT). We noticed that baseline depressive symptoms of most of the participants enrolled in this study were roughly mild-to-moderate (HDRS = 17.26 ± 5.52 for the pioglitazone group, HDRS = 13.94 ± 4.03 for the placebo group; data presented as mean ± standard deviation), which may affect amounts of reduction in HDRS scores to some extent. Another study examining the predictive role of peripheral leukocyte telomere length (LTL) in the antidepressant effect of PPAR-γ agonists was conducted by Rasgon *et al.* [162]. Medically stable patients with non-remitted depression were treated with adjunctive pioglitazone (30 mg/day) or placebo in addition to treatment-as-usual for 12 weeks. Clinical improvements were assessed by OGTT, LTL measurements, and HDRS. The result showed that patients with longer baseline telomeres exhibited greater declines in depression severity in the pioglitazone arm, but not in the placebo arm.

Metformin is a typical biguanide drug acting through multiple pathways, including enhancing insulin sensitivity, reducing hepatic glucose output, and limiting intestinal glucose reabsorption [158, 163]. In a clinical trial performed by Abdallah *et al.* [164], it was used as an adjunct to antidepressants for MDD. Adult outpatients with MDD (DSM-IV criteria, HDRS > 18) were randomly separated into groups that received fluoxetine (20 mg once daily) plus metformin 1000 mg or matching fluoxetine plus placebo for 12 weeks. Depressive symptoms were assessed by HDRS score at weeks 0, 4, 8, and 12, while several serum biomarkers were measured before and after therapy. Improvements in HDRS total scores were significantly higher in the metformin group than in the placebo group after 4, 8, and 12 weeks of treatment. Also, response and remission rates were significantly higher in the metformin group. Furthermore, based on the detection of serum biomarkers, the authors attributed these clinical improvements in the metformin group to its anti-inflammatory, antioxidant, and neuroprotective effects. It should be noted that participants who met the metabolic syndrome criteria were excluded from this study.

### 3.5 Lipid Regulating Agents with Anti-inflammatory Properties

Statins (3-hydroxy-3-methylglutaryl-coenzyme A (HMG-CoA) reductase inhibitors) are among the most prescribed medications worldwide [165]. Basically, they are frequently used to treat dyslipidemia and cardiovascular diseases [166]. More recently, they have been reported to possess immunomodulatory and anti-inflammatory properties [167], and have been investigated for potential antidepressant effects. Santanello *et al.* [168] evaluated, through CES-D scales, the effect of administering lovastatin on older patients aged ≥ 65 years. In this study, participants with LDL level ranging from 159 mg/dL to 221 mg/dL were randomly assigned to receive diet plus placebo, diet plus 20 mg/day of lovastatin, or diet plus 40 mg/day of lovastatin. Assessments regarding health-related quality of life, including CES-D, were completed at baseline and 6 months post-treatment. The results showed no statistically significant differences between treatment groups in terms of changes in CES-D scores and other health-related quality-of-life measures. Ghanizadeh and Hedayati [169] performed another trial to investigate the augmentation of fluoxetine with lovastatin for treating MDD. This study included 61 patients with MDD who met the DSM-IV criteria and allowed them to receive treatment with fluoxetine (up to 40 mg/day) plus lovastatin (30 mg/day) or matched fluoxetine plus placebo. Depressive symptoms were evaluated using HDRS at baseline, week 2, and week 6. During the treatment period, HDRS scores significantly decreased in both groups, while in the lovastatin-combination group, they decreased more than placebo at the end of the trial.

Stewart *et al.* [170] conducted a 4-year trial to assess the effect of long-term reduction of serum cholesterol with pravastatin sodium on psychological well-being. There was no significant difference in depression found between pravastatin (40 mg/day) and placebo treatment. The results were obtained by evaluating the general health questionnaire (GHQ) and calculating “depression cases” based on GHQ scores. Carlsson *et al.* [171] evaluated, through the 15-item Geriatric Depression Scale (GDS-15), the effect of receiving pravastatin and tocopherol (vitamin E) alone and in combination, on health-related quality of life in older adults. The enrolled subjects were divided into two groups: group 1 received pravastatin for 6 months and then pravastatin plus tocopherol for another 6 months; group 2 received tocopherol for 6 months and then pravastatin plus tocopherol for an additional 6 months. After 6 and 12 months, there were no significant changes found in depression or other measures of health-related quality of life in either group.

The study by Gougol *et al.* [172] included patients diagnosed with MDD who fulfilled the DSM-IV TR criteria with an HDRS score ≥ 25. The simvastatin group received fluoxetine (up to 40 mg/day) plus simvastatin (20 mg/day) for 6 weeks, while the placebo group received matching fluoxetine plus placebo. Evaluations were performed using HDRS at baseline and weeks 2, 4, and 6. The results of this study were positive, showing that depressed patients treated with adjuvant simvastatin had significantly greater reductions and early improvements in HDRS scores and higher response rates compared to placebo. However, another study conducted by Chan *et al.* [173] suggested that high-dose simvastatin treatment for 24 months significantly increased depression as determined by the HRDS score in patients with secondary progressive multiple sclerosis. But this result was not statistically different between the simvastatin and placebo-treated groups.

According to the randomized, double-blind clinical trial performed by Haghighi and collaborators [174], adjuvant atorvastatin improved depressive symptoms and blood lipid values in patients suffering from MDD. In this study, the diagnosis of MDD was according to DSM-V criteria, while the improvement was evaluated by HDRS. Only patients with an HDRS score ≥ 25 were included and assigned to receive atorvastatin (20 mg/day) or placebo in addition to the standard medication of 40 mg/day citalopram. Furthermore, Abbasi *et al.* [175] conducted a trial to compare the probable antidepressant effects of simvastatin and atorvastatin in post-coronary artery bypass graft (CABG) patients. Individuals aged 18-50 years with a history of CABG within the past 6 months and who met DSM IV-TR criteria for the diagnosis of MDD with an HDRS score of ≤ 19 were included in the study. They were randomized to receive treatment with either simvastatin (20 mg/day) or atorvastatin (20 mg/day) for 6 weeks. Improvements in depression were assessed by HDRS at baseline, week 3, and week 6. The results demonstrated a greater improvement in depressive symptoms in the simvastatin group than in the atorvastatin group throughout the treatment course without any serious adverse effect. Moreover, patients receiving simvastatin showed a faster response to treatment compared to the atorvastatin group.

### 3.6 Glucocorticoids and Endogenous Substances

Early in 1995, Arana *et al.* [176] published a trial that assessed the effectiveness of dexamethasone for the treatment of depression. Briefly, thirty-seven outpatients diagnosed with MDD according to DSM-III-R criteria (also HDRS > 20) were randomly assigned to receive either placebo or 4 mg/day of oral dexamethasone for 4 days. Baseline HDRS scores were compared to scores obtained 14 days after the first dose of the study drug. “Response” was identified as a reduction in HDRS score ≥ 50% from baseline or a terminal HDRS scale score ≤ 14. Eventually, the frequency of response of the dexamethasone group was found to be significantly greater than the percentage of the placebo group. Similarly, DeBattista *et al.* [177] conducted another study to examine the acute antidepressant effects of intravenous hydrocortisone and ovine corticotropin-releasing hormone (CRH) infusions in patients with MDD who met DSM-III-R criteria. The recruited patients were randomized into groups receiving intravenously 1 µg/kg of ovine CRH, 15 mg of hydrocortisone, or saline in a double-blind fashion on day 1. HDRS assessments were completed before the study and the day after the treatment. The results exhibited that the improvement in depression scores in the hydrocortisone group was significantly higher than that in the ovine CRH group and placebo group. However, the findings may be biased due to the short interval between the two HRDS assessments. Palmitoylethanolamide (PEA), an active endogenous compound naturally existing in the CNS, is an endocannabinoid-like lipid mediator with anti-inflammatory, analgesic, antimicrobial, immunomodulatory, and neuroprotective effects [178]. To evaluate the efficacy and tolerability of PEA add-on therapy for depression, Ghazizadeh-Hashemi *et al.* [179] performed a study including 54 patients with MDD (DSM-V) and an HDRS score ≥ 19. These subjects were randomly allocated into groups receiving either PEA (600 mg bid) or placebo in addition to citalopram (40 mg/day) for 6 weeks. Depressive symptoms were evaluated using HDRS at baseline and weeks 2, 4 and 6. Compared to the placebo group, patients treated with PEA plus citalopram demonstrated a significantly greater reduction in HDRS scores from week 2 to week 6. Moreover, at the end of this trial, the PEA group also had a higher rate of response, not remission, than the placebo group.

### 3.7 Other Anti-inflammatory Approaches

Since the pathogenesis of MDD involves intricate pathophysiological mechanisms, complementary and alternative medicine (CAM) is widely used in its treatment [180]. In addition to the above-mentioned drugs, there are many other anti-inflammatory methods for treating major depression. Many Chinese herbal medicine (CHM) formulations and single Chinese herbs have been used to treat depression. A systematic review by Yeung and collaborators [181] combined the previous trials and evaluated the efficacy, safety, and type of CHM for major depression. It showed that the most frequently used formulas were Xiaoyao decoction, Chaihu Shugan decoction and Ganmai Dazao decoction, while Chaihu (*Bupleurum chinense* DC.), Baishao (*Paeonia lactiflora* Pall.) and Fuling (*Poria Cocos (Schw.)* Wolf.) were the most commonly used single herbs. Besides, Gancao (*Glycyrrhiza uralensis* Fisch.) is the shared ingredient in all three of the formulas [181]. Specifically, Jianghuang (*Curcuma longa* L.) is known for its anti-inflammatory effects by modulating NF-κB and reducing pro-inflammatory cytokines, as well as for its antioxidant effects [182]. Curcumin, a polyphenolic compound, is the main constituent of this CHM. At least 10 clinical trials have been conducted to evaluate its antidepressant effects [183-191]. Despite differences in doses, formulations, or adjuvants, the combined results suggest that curcumin is effective in improving depression in humans [182]. Likewise, glycyrrhizic acid (GZA, the main component of Gancao) has been recently reported to improve symptoms in depressed patients (especially baseline CRP > 3 mg/L) better than placebo when used in combination with SSRIs. A reduction in TNF-α partially mediated the association between GZA treatment and clinical improvement [192].

Although pharmacotherapy remains the primary treatment modality for MDD, given its anti-inflammatory properties, lifestyle modification has emerged as a potential non-pharmacological anti-depressive approach that has been studied in clinical practice [7]. First, in terms of dietary strategy, several nutrients, foods, or dietary supplements with anti-inflammatory effects have been examined for treating MDD. Meta-analyses of clinical findings suggest that moderate consumption of fish and other food of “high quality” is linked to a lower risk of depression, especially in women [193, 194]. These foods, especially oily fishes as well as fish oil, are rich in ω-3 polyunsaturated fatty acid (PUFA), particularly docosahexaenoic acid (DHA; 22:6 ω-3) and eicosapentaenoic acid (EPA; 20:5 ω-3). In-depth studies demonstrate that EPA, but not DHA, is responsible for the most antidepressant effects of ω-3 PUFA [195, 196]. Apart from seafood rich in ω-3 PUFA, fruits and vegetables with adequate vitamins, beverages containing sufficient polyphenols, probiotic foods that module gut microbiota, and a Mediterranean diet with a low dietary inflammatory index are also promising antidepressant dietary options [197]. Second, physical exercise has been shown to enhance brain function and reduce neurodegeneration by enhancing neuroplasticity, as well as modulating neuroinflammation and microglial activation, and is therefore used in the treatment of neurological and psychiatric diseases [198, 199]. Accumulating meta-analytic studies pooling clinical trials suggest that exercise moderately improves major depressive disorder and depressive symptoms [200, 201], whether as monotherapy, adjunctive therapy, or combination therapy [202]. However, exercise regimens are often highly heterogeneous, with different types, intensities, durations, and frequencies. Third, as an effective stress relieving method, the effects of mindfulness-based interventions (MBI) on cytokine and cortisol levels have been studied, and there are promising data supporting that interventions significantly reduce inflammation [203, 204]. In fact, many trials have already been conducted to evaluate the effectiveness of MBI, including mindfulness-based stress reduction (MBSR), MBCT, and mindfulness meditation (MM), in improving MDD. In general, these mindfulness-based therapies are effective in reducing depressive symptoms and depression relapse [205, 206]. As Johannsen and colleagues [207] concluded in a meta-analysis, putative mediators of acceptance and mindfulness-based therapies, including mindful attention, decentering, and acceptance, mediated treatment effects on anxiety and depression. Current evidence also suggests that mindfulness can treat depression by targeting the very inflammatory pathways associated with a worse prognosis in cancer [203]. Collectively, countless clinical studies have demonstrated the anti-inflammatory effects of lifestyle changes, such as exercise, diet, and MBI in the context of depression and other psychological issues. However, most of them are less evidence-based (especially with substantial methodological flaws) compared to pharmacological treatments [7]. Clinicians and patients still need to be cautious when applying these non-pharmacological therapies.

## 4 DISCUSSION

This article has briefly introduced the rationale of anti-inflammatory therapy for depression and provided a comprehensive review of the current clinical evidence for anti-inflammation-targeted therapies for depression. The results suggest that anti-inflammatory therapy can effectively improve depressive symptoms under certain conditions, whether as a monotherapy or as an add-on therapy. Specifically, it is mainly manifested in the following aspects: (1) Promoting the reduction of rating scale scores in patients with MDD, and improving the response and remission rate; (2) Hastening the onset of traditional serotonergic antidepressants; (3) Improving depressive symptoms in patients with somatic diseases, such as inflammatory diseases, metabolic disorders, and tumors. However, these results should be implemented with caution. There is still much confusion with respect to the application of anti-inflammatory therapy for MDD. These studies also suggest that routine diagnosis should be complemented with the assessment of biological factors, such as inflammatory biomarkers, to identify effective treatments for depression, including anti-inflammatory drugs. Moreover, considering the lack of evidence to support the standard regimens of anti-inflammatory therapy, issues including drug dosage, the timing of dosing or withdrawal, and assessment tools, still need to be explored.

### 4.1 Anti-inflammatory Treatment as an Adjuvant Therapy

Generally, when conventional antidepressant monotherapy fails to produce a satisfactory response to depressive symptoms, in addition to switching drugs, augmenting the antidepressant effect with an adjunctive treatment modality is a common strategy. During the past few decades, many reports have linked depression with inflammation, cytokines, or neuroinflammation, but few high-quality clinical trials are devoted to examining the effectiveness, efficiency, efficacy, and safety of anti-inflammatory treatment for MDD. Kohler *et al.* [208] conducted a meta-analysis study based on 14 trials, 10 of which evaluated the use of NSAIDs and 4 that examined cytokine inhibitors. The combined effect demonstrated a robust result that anti-inflammatory treatment reduced depressive symptoms compared to placebo in patients with MDD and depressive symptoms. In another meta-analysis examining the efficacy of anti-inflammatory treatment on depression by Kohler and collaborators [106], 36 random clinical trials involving 6 classes of drugs were identified and included. The results confirmed that anti-inflammatory agents as add-ons improved depressive symptoms compared to placebo in MDD patients. Also, the combined use of anti-inflammatory with conventional therapy significantly improved response and remission rates. Further, Adzic *et al.* [209] comprehensively discussed the utility and inadequacy of antidepressant strategies focused on inflammatory pathways based on previous preclinical and clinical findings. They concluded that SSRIs exhibited a strong beneficial effect in restraining inflammatory cytokines in depression, while different anti-inflammatory agents exerted antidepressant effects *via* different mechanisms. Adjunctive use of anti-inflammatory treatment is a feasible augmentative therapy for patients with moderate-to-severe depression. Kopschina Feltes *et al.* [210] and Shariq *et al.* [211] hold the same viewpoints. They also suggest that serum levels of inflammatory biomarkers (primarily IL-1β, IL-6, TNF-α, and hs-CRP) in depressed patients are useful biomarkers that can be used in combination with the assessment of depression scores.

In terms of treatment goals, we expect that the application of anti-inflammatory therapy for MDD is supposed to achieve: (1) A stronger effect. It is widely known that the commonly used traditional monoaminergic antidepressants only achieve a 67% remission rate after 4 steps of treatment [5]. Two-thirds of patients with depression will not remit after initial antidepressant treatment [212], while only half of the patients have significant clinical responses after taking antidepressants [213]. Therefore, the urgent expectation at this stage is that anti-inflammatory therapy should substantially strengthen the response and remission rate of these traditional drugs. Nonetheless, we do not insist that an anti-inflammatory strategy is the only way to help achieve this goal. Other augmentative options may include regular aerobic exercise, good social support, appropriate psychotherapy, healthy diet and supplements, *etc*. [28] (2) A faster onset. The traditional monoaminergic antidepressants commonly used at present have relatively a delayed response and an onset of action ranging from hours to days, even up to weeks [214]. Apart from anti-inflammatory drugs, ketamine and its derivates have been reported to have a rapid onset followed by sustained action mediated by a complex multi-target mechanism, including modulation of inflammation [215, 216] and upregulation of BDNF [214]. (3) A higher acceptance. On one hand, many current antidepressants have drug-related side effects, including headache, nausea or vomiting, agitation, sedation, and sexual dysfunction, which may affect patient tolerance and compliance [217]. On the other hand, the heavy use of antidepressants imposes an enormous economic burden on patients and public health services. The annual total expenditure for antidepressants has been reported to be approximately US$17.4 billion in the US [218]. Moreover, TRD patients involve significantly higher healthcare resource utilization, translating into higher average total costs (US$21,015(TRD) *vs.* US$14,712(No TRD)) and MDD-related costs (US$1201(TRD) *vs.* US$471(No TRD)) compared to non-TRD patients [219]. Besides, developing a new antidepressant drug is also expensive for pharmaceutical companies.

### 4.2 Problems to be Solved before Clinical Practice

From our point of view, there are still the following problems to be solved in the anti-inflammatory treatment of depression, which are related to stratified and personalized treatment.

#### 4.2.1 Who will be Included in the Anti-inflammatory Treatment Attempt?

As mentioned earlier, the majority of clinical trials of anti-inflammatory therapy for depression recruit participants based on rating scale scores without adequate consideration of physiological indicators, and there are indeed no biomarkers to rely on for the diagnosis of depression at present. Further, many of the early studies (especially those investigating cytokine inhibitors) included patients with comorbid diseases, such as inflammatory diseases [110, 111, 115, 117-119, 121, 123-125]. In studies specifically targeting MDD, sample sizes were generally small (about 30 subjects per arm), and multicenter studies were lacking. Moreover, since the prevalence of depression in women is about twice as high as in men [220], there may be gender differences in the pathophysiology of depression onset and development. Accordingly, the effect of anti-inflammatory treatment may also vary by gender [132]. Besides, among the patients diagnosed with depression according to DSM criteria, we should consider the type (relapse or first episode, drug-naïve or treatment-resistant) and severity (mild, moderate, or severe) of the patients who will receive anti-inflammatory therapy. Based on the studies reviewed in this article, the treatment effects of mild/moderate and severe MDD seem to be inconsistent [147, 148]. Meanwhile, the subjects of recent studies are mainly patients with TRD [136, 150, 151].

#### 4.2.2 What Treatment Regimen will the Participants Receive?

Most of the studies included in this article used combination therapy, where anti-inflammatory agents were given as an adjunct to treatment as usual (including antidepressants, mood stabilizers, and antipsychotics, as well as psychotherapy and other psychosocial interventions) [149, 162]. However, considering comorbidities and clinical ethics, cytokine inhibitors are currently more often used as monotherapy [110, 111, 113, 115, 118, 119, 121, 123-125]. In terms of which anti-inflammatory intervention/drug should be selected, the current research results still cannot answer this question. Considering the complex interconnection of biological functions of different cytokines, these inflammatory mediators are usually defined as networks. So it may be easier to understand that selective anti-inflammatory agents can not only cause significant changes in specific target molecules, but also affect other components in this network. Since it is not yet determined which cytokine is the most crucial therapeutic target, the application of one or more safe, stable, economic, and effective broad-spectrum anti-inflammatory drugs may be an acceptable option at this stage. As for the dosage, the currently recommended dosages of anti-inflammatory drugs for MDD are mainly based on the dosages used to treat inflammatory diseases. For example, the suggested dose of celecoxib is mostly 200 mg bid (400 mg/day) [130-133, 135, 140-142]. However, studies on the dose-effect relationship are scarce.

#### 4.2.3 How to evaluate the efficacy of anti-inflammatory treatment?

According to the included literature, most studies still use symptomatic rating scales, either self-rated or examiner-rated, to evaluate the effect of anti-inflammatory therapy on depression. However, there may be ceiling/floor and concealed effects in these trials due to the heterogeneity in the mean baseline depression scores of the patients. In a meta-analysis of placebo-controlled randomized clinical trials of antidepressants, Maslej *et al.* [221] included 91 eligible trials with a total of 18,965 participants. As a result, they found no evidence that variability in observed response (as measured by total depression scores) among participants receiving antidepressants was greater than among those receiving a placebo. Hence, they suggest that future studies explore whether individual symptom scores or biomarkers are associated with variability in response to antidepressants. Despite hs-CRP, emerging research on the role of neutrophil-to-lymphocyte ratio (NLR), platelet-to-lymphocyte ratio (PLR), and monocyte-to-lymphocyte ratio (MLR) in depression also suggests NLR to be significantly associated with depression, and that it can be used as an indicator of depression [222-224].

In addition to the above concerns, there are other issues that need to be addressed. For example, is the mechanism by which anti-inflammatory drugs act as antidepressants by modulating inflammation a direct (or an indirect) effect? If so, which one is their target, central or peripheral inflammation? This may determine whether drug candidates should have the ability to penetrate the blood-brain barrier. Some studies have speculated that the reason why patients with high inflammation are more responsive to this therapy is that they have insufficient BBB integrity [21, 95]. Another key question is: among the present candidate molecules, whether there are specific targets of inflammation leading to depression, which can be used for diagnosis and treatment? As mentioned earlier, different inflammatory factors have been found to be closely related to depression and are presumed to be biological targets of depression. However, there is still insufficient evidence to support the effectiveness of any single target [225]. In order to improve the sensitivity and specificity of inflammatory indicators, we have proposed to use a biomarker panel instead of a single/specific one [226]. However, this is just an idea based on previous research and has not been verified by large-scale clinical research. A major obstacle at this stage is that there are still disputes about which biomarkers should be included. We believe that, in the future, the wide application of big data and the rapid progress of medical testing technology may accelerate the establishment of this panel.

### 4.3 Insights from Basic/Preclinical Research

It is not difficult to understand from the above evidence that the treatment methods based on the inflammation hypothesis of depression highly address the adjustment of the immune system. In addition to these methods that have already been applied in clinical trials, the rapid progress in basic and preclinical research also provides new targets. Here, we have briefly summarized several representative and prospective treatment conceptions close to translational applications.

#### 4.3.1 Switching of the Microglial Activation Phenotype

As immune cells are stationed in the CNS, microglia can sense and react to danger signals, thus affecting neurogenesis and depression-like behavior. Despite the controversy, the classification of M1 and M2 subtypes represents different functional states of microglia. Under stress, microglia are induced towards the M1 type, releasing inflammatory factors and causing a neuroinflammatory reaction. After the inflammation subsides, microglia shift towards the alternative activated M2 phenotypes, which is responsible for neuroprotection [227-229]. Therefore, the switching of microglia activation phenotype is a possible treatment for depression, especially in terms of reducing the M1 phenotype and increasing the M2 phenotype [230]. It has been shown that pretreatment of anti-inflammatory IL-10 before LPS administration can prevent the expression of pro-inflammatory cytokines, TNF-α, IL-6, and COX-2 [231]. Besides, glatiramer acetate (GA) is a promising molecule that can alter the inflammatory environment by recruiting Th2 T cells into the CNS, induce the production of another anti-inflammatory cytokine IL-4, and improve depressive-like behavior [232]. More interestingly, there is evidence that PPAR-γ can inhibit the activation of microglia, promote M2 polarization, and inhibit inflammatory cytokines, which is considered to be a major reason why PPAR-γ agonists can play an anti-inflammatory and antidepressant role [233]. Similarly, minocycline is a microglia activity inhibitor that can selectively prevent M1 microglia from polarization into a pro-inflammatory state [234]. In the future, even allogeneic blood exchange and mesenchymal stem cell transplantation may become options for transforming microglia.

#### 4.3.2 Control of the TLR4-mediated Sterile Inflammation

Toll-like receptors (TLR) are a major class of receptors for detecting DAMP and PAMP. It is essential for the innate immune response and sterile inflammation of the CNS associated with depression [6, 80]. Animal studies have shown that TLR4 plays a key role in regulating neuroinflammation and depressive-like behavior [235]. Upon ligand recognition, TLR4 activates glycogen synthase kinase-3 (GSK3) that activates NF-kB to promote the production of proinflammatory cytokines [236]. Recently, some TLR4 ligands have been found to participate in depressive-like behavior, including HMGB1, myeloid-related protein 8/14 (Mrp8/14, also known as S100A8/9), and adenosine triphosphate (ATP) [237-240]. Different strategies can be adopted to alleviate depressive-like behavior by regulating the release and function of these alarmins. For example, minocycline can inhibit the activation of microglia in mice, reduce the release of HMGB1 from microglia and neurons, and alleviate the behavioral and cognitive deficits caused by chronic stress [241]. Ethyl pyruvate (EP) may inhibit the acetylation and release of HMGB1 by affecting the sirtuin1/signal transducer and activator of the transcription (SIRT1/STAT) pathway, and ultimately improve the depressive-like behavior of mice [242]. Treatment of mice with GZA, the natural inhibitor of HMGB1, can ameliorate depressive-like behavior by subsequently regulating the key enzymes of the kynurenine pathway [243]. Furthermore, TAK-242, a selective inhibitor of TLR4, can significantly block the increase in TNF-α protein and the decrease in myelin basic protein induced by HMGB1 in hippocampi, thus improving the depressive-like behavior of experimental mice [244]. Additionally, the Mrp14 inhibitor ABR-215757 effectively restores depressive symptoms and TLR4/NF-κB signaling activation induced by chronic stress [238].

#### 4.3.3 Inhibition of the NLRP3 inflammasome overaction

The important role of IL-1β has already been introduced in this review. In fact, its production and maturation are controlled by the multi-protein complex called inflammasome, which consists of the nucleotide-binding oligomerization domain (NOD)-like receptor (NLR) family, the effector protease caspase-1, and the adapter apoptosis-associated speck-like protein containing a caspase recruitment domain (ASC). Of all inflammasomes, the NOD-, leucine-rich repeat (LRR)-, and pyrin domain (PYD)-containing protein 3 (NLRP3) inflammasome has been studied extensively and proposed to be a potential target for the treatment of depression [245, 246]. We have provided the first preclinical evidence linking the NLRP3 inflammasome to depressive symptoms and verified the result by gene knockout [247, 248]. There are also many research reports on the treatment of depression by targeting the NLRP3 inflammasome. Firstly, because caspase-1 is the effector organ responsible for cleaving pro-IL-1β, its inhibitors, such as Ac-YVAD-CMK and VX765, can improve the depressive-like behavior of mice [247, 249]. Secondly, the application of NLRP3 inhibitors, such as MCC950 and glibenclamide, unexpectedly prevents inflammation-related depressive-like behavior [250-252]. Thirdly, regulating the upstream molecule of NLRP3 inflammasome activation also yields an antidepressant-like effect. Stress-induced depressive-like behavior can be eliminated by P2X7-gene knockout and chronic administration of P2X7 antagonists, such as bright blue G (BBG), A438079, and JNJ-55308942 [253-256].

### 4.4 A Subtle Change in Clinical Thinking

**Fig. (1):**
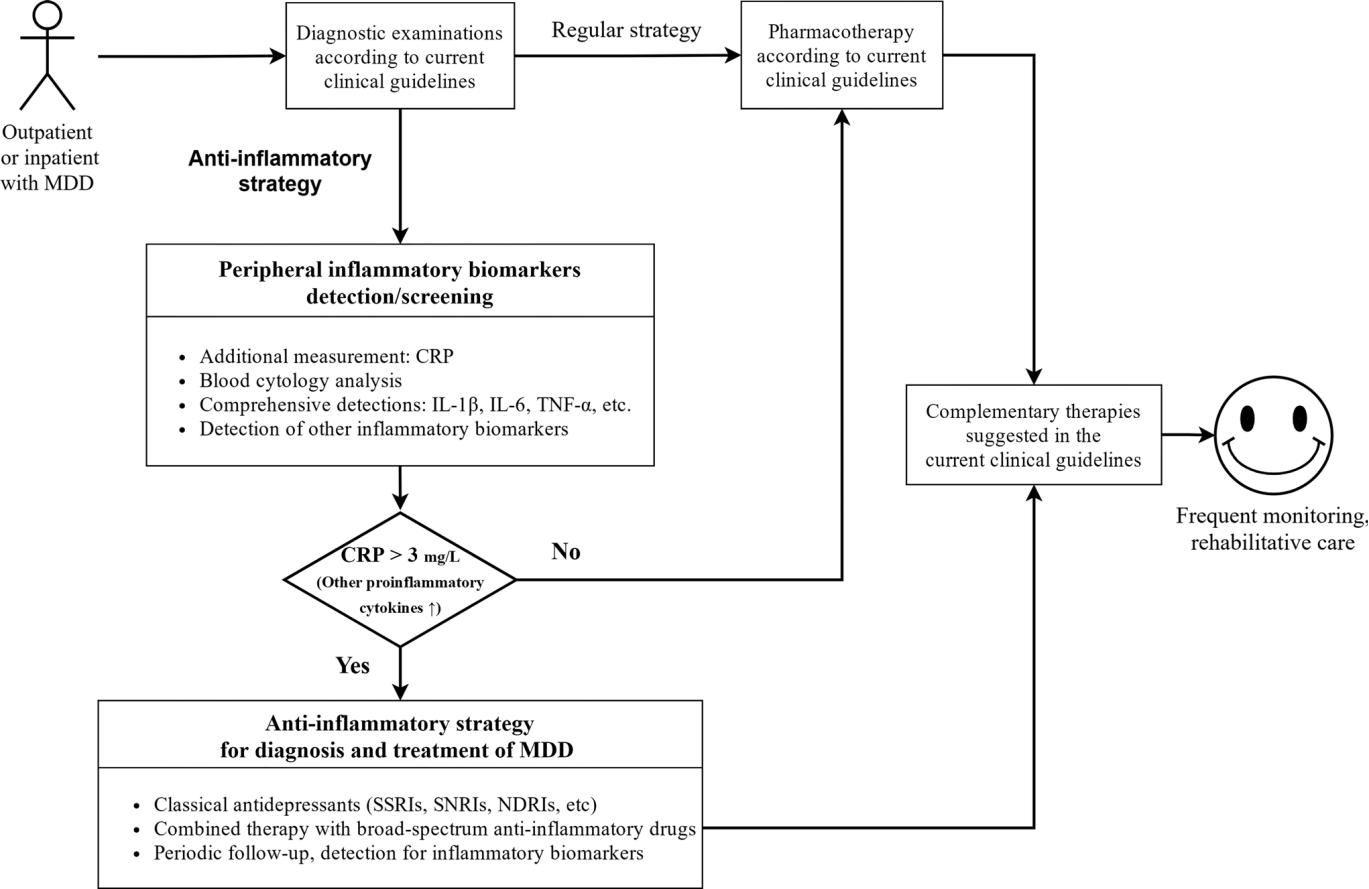
Flowchart of novel anti-inflammatory strategy for the diagnosis and treatment of depression. Regularly, the outpatients or inpatients who meet the symptomatic diagnostic criteria for major depressive disorder will receive pharmacotherapy (primarily monoaminergic antidepressants) and complementary therapies suggested in the current clinical guidelines. In the novel anti-inflammatory strategy, these patients may receive additional inflammatory assessments associated with depression. These indicators mainly include serum hs-CRP levels, blood cytology analysis (routine blood test), serum IL-1β, IL-6, TNF-α and other inflammatory cytokines if needed. Based on these screening results, if the patient is classified to be “high-inflammatory”, they were recommended to receive augmentative treatment with broad-spectrum anti-inflammatory agents in combination with treatment as usual. It is worth noting that, in addition to the conventional evaluation methods, we recommend adding continuous monitoring of changes in inflammatory markers during the follow-up visits in the evaluation of efficacy and prognosis of treatment. (**Abbreviations:** MDD, major depressive disorder; hs-CRP, high sensitive C-reaction protein; IL-1β, interleukin-1β; IL-6, interleukin-6; TNF-α, tumor necrosis factor-α; SSRIs, selective serotonin reuptake inhibitors; SNRIs, serotonin noradrenaline reuptake inhibitors; NDRIs, norepinephrine-dopamine reuptake inhibitors).

Based on the cytokine hypothesis of depression and our preclinical [216, 238, 242, 250, 257] and clinical [192] findings, coupled with the complex interaction between different cytokines, we have previously proposed a theory of cytokine-network pathogenesis in depression [12]. This theory implies that both specificity and sensitivity should be emphasized in the selection of biomarkers for the diagnosis and treatment of depression. That is to say, biomarkers that are more consistently altered across animal models and patients should be preferentially considered. In terms of the selection of therapeutic drugs, it may be a wise choice to use safe, effective, and affordable “old drugs” as adjuvants. In line with this theory, we have developed a novel anti-inflammatory strategy for the diagnosis and treatment of depression (Fig. **1**), and provided suggestions for upgrading the current clinical diagnosis and treatment procedures for depression: (1) Detecting serum inflammatory biomarkers, such as hs-CRP and blood cytology analysis, at the first admission of MDD patients (cytokines, including IL-1β, IL-6, and TNF-α may be comprehensively detected if possible), so they can be identified and classified accordingly; (2) Patients with high inflammation at baseline are suggested to receive combined therapy with broad-spectrum anti-inflammatory drugs. According to the current clinical availability and feasibility, we recommend CRP >3 mg/L as a cut-off value. However, this value has not been widely verified by clinical trials, and it may be premature to use it as the gold standard for inflammatory intervention in clinical practice. Therefore, we only suggest that this value be used for further exploratory research in the future. (3) In order to evaluate the efficacy and predict the prognosis, we should also refer to inflammatory indicators instead of paying too much attention to the rating scales.

To sum up, anti-inflammatory therapies have a long way to go before they can be widely used in the clinical practice for treating depression as standard treatment. One important reason is that, as Drevets *et al.* [258] demonstrated, it is still difficult to identify whether the depressive symptoms of an MDD patient are caused or exacerbated by inflammation. Therefore, the problem is not to determine the potential anti-inflammatory treatment, but to identify the patients who may benefit from this therapy. Our abovementioned strategy is mainly based on the statistical difference between the immunological characteristics of patients with depression and those without depression. From the perspective of the large-scale application, blood biomarkers are more accessible and the detection methods are more mature, and can provide sufficient mechanistic information regarding the circulating compartment of the peripheral immune system. In addition to the CRP, immune cell count and cytokine level we have mentioned, whole-genome gene expression and quantitative polymerase chain reaction (qPCR) levels of specific mRNAs [259, 260], and cytokine or gene expression levels stimulated by LPS *in vitro*, may also help to better profile the immune function [261, 262]. However, the extent of central immune function that can be inferred from peripheral immune markers is still unclear. It is necessary to change our clinical thinking. If accurate enough, a patient selection method based on a clinical phenotype will be more convenient and economical than a biological measurement [258]. That is to say, the classic behavioral and symptomatological scales currently used require a substantial revision to distinguish subpopulations of depressive patients more effectively. The development and adoption of immuno-targeted therapy for depression seem to upgrade the diagnosis and stratification principles of MDD and focus instead on transdiagnostic symptom dimensions [258, 263].

### 4.5 Limitations

This review has several limitations. First, considering the search strategy and screening criteria, we may have omitted retrospective studies and some clinical trials that had not taken anti-inflammatory therapy as the primary study design. Second, the antidepressant effects of some drugs not included in this review may also be related to anti-inflammatory effects, but these may only have been found in exploratory analyses and were not the primary outcome. Third, as this is a narrative review, we did not further perform a quantitative and qualitative analysis of the included literature. This part has already been conducted by other studies. Fourth, we have not paid enough attention to side effects, which are reported in only a few studies and are incomplete, and further research is needed.

## CONCLUSION

Based on current clinical evidence, anti-inflammatory therapy is an effective and promising treatment for depression. However, there is still a long way to go to move anti-inflammatory therapy from clinical trials to practical guidelines. The next step toward optimizing treatment efficacy should involve upgrading the diagnostic criteria for major depressive disorder and assessment of responses to antidepressants based on specific symptom profiles, sets of biomarkers, or a combination of them. Research on etiology and pathophysiological mechanisms also needs to be explored continuously and thoroughly to provide more options for translational applications. The novel strategy for the anti-inflammatory therapy of major depressive disorder presented in the current paper may have implications for clinicians and researchers.

## Figures and Tables

**Table 1:** Summary of clinical trials of anti-inflammatory agents for depression.

**Category**	**References**	**Drug**	**Study Design**	**Participants**	**Intervention**	**Instruments**	**Results**
Cytokine inhibitors	Loftus *et al.*, 2008 [110]	Adalimumab	Phase III, double-blind randomized, clinical trial	492 patients aged 18-75 years with moderate to severe Crohn’s disease	Baseline subcutaneous injection with 80-mg adalimumab, followed by a 40-mg dose at week 2, then receiving adalimumab 40 mg every other week (n = 169), adalimumab 40 mg weekly (n = 155), or placebo injections (n = 168) for 52 weeks	ZDS	After induction therapy, patients who received adalimumab maintenance therapy at 40 mg every other week and weekly both reported less depression compared with placebo group
	Kekow *et al.*, 2010 [117]	Etanercept, Methotrexate	Double-blind, randomized clinical trial	528 patients with active early rheumatoid arthritis	Treatment with etanercept 50 mg once weekly plus methotrexate (n = 265) or methotrexate alone (n = 263) for 52 weeks	HADS	No statistically significant difference in the improvement of HADS anxiety and depression subscale scores between groups
	Menter *et al.*, 2010 [111]	Adalimumab	Double-blind randomized, placebo-controlled trial	147 anti-TNF therapy-naïve and topical therapy uncontrolled patients with moderate to severe psoriasis	Treatment with adalimumab 40 mg every other week (n = 44) or matched placebo (n = 52) for 12 weeks	ZDS	Compared with the placebo group, the adalimumab group experienced more reduction in ZDS score after treatment for 12 weeks
	Raison *et al.*, 2013 [113]	Infliximab	Double-blind, randomized, placebo-controlled trial	60 outpatients aged 25-60 with treatment-resistant depression who were on a consistent antidepressant regimen or off medication ≥ 4 weeks	Three infusions of infliximab (5 mg/kg) (n = 30) or placebo (n = 30) at baseline and weeks 2 and 6	HDRS-17	Infliximab did not show generalized efficacy in treatment-resistant depression. Secondary analysis indicated that infliximab had a better improvement in patients with baseline hsCRP > 5 mg/L, while placebo was the opposite
	Tyring *et al.*, 2013 [118]	Etanercept	Double-blind, randomized, placebo-controlled study	121 patients with moderate-to-severe plaque psoriasis with scalp involvement	Group A: Subcutaneous (SC) injection with 50 mg etanercept twice weekly (biw) for 12 weeks, followed by 50 mg etanercept and placebo once weekly (qw) and placebo qw for 12 weeks (n = 59). Group B: Placebo biw by SC injection for 12 weeks, followed by etanercept 50 mg biw for 12 weeks (n = 62)	Patient-Reported Outcomes Measurement Information System (PROMIS) Emotional Distress/ Depression	Patients received treatment with etanercept during the first 12 weeks experienced greater improvements than the other group in emotional distress/depression. Besides, patients who switched from placebo to etanercept got better scores of emotional distress/depression at week 24 than week 12
	Simpson *et al.*, 2016 [124]	Dupilumab	Double-blind, randomized, placebo-controlled, parallel-group dose-ranging study	379 adult patients with moderate to severe atopic dermatitis	Subcutaneous treatment with dupilumab 300 mg once weekly (qw) (n = 63), 300 mg every 2 weeks (q2w) (n = 64), 200 mg q2w (n = 61), 300 mg q4w (n = 65), or 100 mg q4w (n = 65), or placebo qw (n = 61)	HADS	Patients treated with dupilumab of all doses mentioned in the study reported significant improvements in both reductions of HADS total scores and depression subscale scores compared with placebo
	Griffiths *et al.*, 2017 [121]	Ixekizumab	Double-blind, randomized, placebo-controlled trial	320 adult outpatients with psoriasis for at least 6 months, comorbid with depressive symptoms as defined by QIDS-SR16 score ≥ 11	Ixekizumab 80 mg every 4 weeks (q4w; n = 120), ixekizumab 80 mg every 2 weeks (q2w; n = 107 9), or placebo (n = 93), initial dose of ixekizumab was 160 mg	QIDS-SR16	Treatment with ixekizumab for 12 weeks showed greater improvement in QIDS-SR16 total scores and remission rates of depressive symptoms compared with placebo in patients with moderate-to-severe plaque psoriasis (psoriasis) and depressive symptoms
	Sun *et al.*, 2017 [119]	Sirukumab, Siltuximab	Double-blind, placebo-controlled trial	Trial 1: 176 methotrexate-resistant RA patients with CRP ≥ 10mg/L. Trial 2: 65 HHV8-negative MCD patients with no prior exposure to IL-6 or IL-6R targeted therapies	Trial 1: Part A, sirukumab 100 mg or placebo or every 2 weeks (q2w) through week 10, with crossover treatment during weeks 12-22; Part B, sirukumab (100 mg q2w, 100 mg q4w, 50 mg q4w, or 25 mg q4w) through week 24, or placebo through week 10 with crossover to sirukumab 100 mg q2w (weeks 12-24). Trial 2: intravenous infusions of siltuximab (11 mg/kg) or placebo every 3 weeks	SF-36	Sirukumab and siltuximab produced significantly greater improvements on depressive symptoms compared with placebo
Cytokine inhibitors	Kim *et al.*, 2018 [123]	Ustekinumab	Open label trial	15 patients aged ≥ 18 years with moderate to severe plaque psoriasis and 15 sex-matched healthy volunteers	Subcutaneous administration of ustekinumab 45 mg (or 90 mg if baseline body weight ≥ 100 kg) and repeated after 4 weeks, followed by every 12 weeks	HDRS, BDI	Treatment with ustekinumab significantly reduced the depressive symptoms, as evaluated using BDI and HDRS
	Cork *et al.*, 2020 [125]	Dupilumab	Double-blind randomized, placebo-controlled, phase 3 trial	1379 patients aged ≥ 18 years and had atopic dermatitis for ≥ 3 years	Subcutaneous dupilumab 300 mg once weekly (qw) (n = 462), or every 2 weeks (n = 457), or placebo qw (n = 460) for 16 weeks	HADS	Both dose regimens of dupilumab treatment improved depressive symptoms in patients with atopic dermatitis at week 2 than placebo, and persisted through week 16, compared with placebo
	Webers *et al.*, 2020 [115]	Infliximab	Ancillary study to a randomized controlled trial	23 adults with ankylosing spondylitis (BASDAI score ≥ 4 and spinal pain assessment score ≥ 4)	Infusion with 5 mg/kg infliximab (n = 16) or placebo (n = 7) at weeks 0, 2, 6, 12, and 18; then both received infliximab therapy from week 24 until week 54	CES-D	Infliximab improved depressive symptoms after 24 weeks in patients with ankylosing spondylitis compared to placebo. After the original placebo group switched to infliximab, the mean CES-D score decreased to the same as the infliximab group at week 54
NSAIDs	Muller *et al.*, 2006 [130]	Celecoxib	Double-blind, randomized, placebo-controlled, parallel-group trial	40 patients aged 23-63 years suffering from an acute depressive episode	Treatment with 4-10 mg reboxetine plus placebo (n = 20) or reboxetine plus 400 mg celecoxib (n = 20) for 6 weeks, reboxetine started with 2 mg for 2 days before 4 mg, celecoxib from 200 to 400 mg/day within 3 days	HDRS-17	The celecoxib group showed significantly greater improvement in HDRS-17 scores compared to the reboxetine-alone group
	Akhondzadeh *et al.*, 2009 [131]	Celecoxib	Double-blind, placebo-controlled trial	40 outpatients aged 24-46 years with MDD (HDRS score ≥ 18), with a score ≥ 2 on item 1 of HDRS	Ingesting fluoxetine 40 mg/day plus celecoxib 400 mg/day (200 mg bid) (morning and evening) (n = 20) or fluoxetine 40 mg/day plus placebo (n = 20)	HDRS-17	The combination of fluoxetine and celecoxib showed a significant superiority over fluoxetine plus placebo in improving depressive symptoms
	Abbasi *et al.*, 2012 [132]	Celecoxib	Double-center, double-blind, randomized, placebo-controlled, parallel-group study	40 patients aged 18-50 years with MDD (HDRS score > 18), with a score ≥ 2 on item 1 of HDRS	Adjunctive celecoxib 200 mg twice daily (n = 20) or matched placebo (n = 20) added to sertraline 200 mg/day (half dose in the first week) for 6 weeks	HDRS-17	The add-on celecoxib group showed significantly greater reduction in HDRS scores as well as more response and remission than the placebo group
	Fields *et al.*, 2012 [140]	Celecoxib, Naproxen	Double-blind, randomized, placebo-controlled, primary prevention trial	2528 cognitively normal participants aged ≥ 70 years with a family history of Alzheimer-like dementia	Monotherapy with celecoxib 200 mg twice daily (bid) (n = 726), naproxen sodium 220 mg bid (n = 719) or matching placebos (n = 1083) for at least 12 months	GDS	Compared with placebo, treatment with celecoxib or naproxen did not improve depressive symptoms in the specific population over time
	Iyengar *et al.*, 2013 [141]	Ibuprofen, Naproxen, Celebrex	Pooled data of 5 double-blind, randomized, placebo-controlled, parallel-group studies	A total of 1497 patients aged ≥ 40 years, diagnosed with active and symptomatic osteoarthritis	Ingesting ibuprofen 800 mg 3 times daily or naproxen 500 mg twice daily (n = 593), or celebrex 200 mg daily (n = 607) or matched placebo (n = 297) for 6 weeks	PHQ-9	NSAIDs usage demonstrated a trend towards reduction of depression symptoms as defined by PHQ-9 in patients with osteoarthritis
	Jafari *et al.*, 2015 [133]	Celecoxib	Single-center, double-blind, randomized, placebo-controlled, parallel-group study	40 patients aged 18-50 years with acute brucellosis and mild to moderate depression due to brucellosis (HDRS-17 score < 19)	Ingesting celecoxib 200 mg capsule (n = 20) or matched placebo (n = 20) twice daily for 8 weeks plus antibiotic therapy for brucellosis (600-900 mg rifampin daily plus 100 mg doxycycline twice daily)	HDRS-17	Adjunctive celecoxib showed greater decrease in HDRS scores and higher response rate compared to placebo from baseline to week 8. Besides, HDRS-17 scores were significantly lower in the celecoxib group than the placebo group at weeks 4 and 8
NSAIDs	Majd *et al.*, 2016 [134]	Celecoxib	Double-blind, randomized, placebo-controlled, parallel-group pilot study	23 female outpatients aged 18-50, diagnosed with first episode of major depression	Ingesting sertraline plus celecoxib 100 mg twice daily (n = 14) or sertraline plus placebo (n = 9) for 8 weeks, 25 mg/day sertraline for the first 3 days, then 50 mg/day, and up to 100 mg/day after 4 weeks if needed	HDRS-17	Add-on treatment with celecoxib showed a greater reduction in HDRS-17 and a higher response rate compared to placebo after 4 weeks, but did not maintain through the end of week 8
	Mohammadinejad *et al.*, 2015 [142]	Celecoxib, Diclofenac	Double-blind, randomized, controlled, parallel-group trial	52 outpatients aged 18-70 years with breast cancer and mild to moderate depression (HDRS score ≤ 18), who suffered from pain and needed analgesics	Receiving either 200 mg celecoxib (200-mg capsule) (n = 26) or 50 mg diclofenac twice daily (50-mg capsules) (n = 26) for 6 weeks	HDRS-17	Greater improvement was observed in the HDRS score of the celecoxib group compared with the diclofenac group
	Mahagna *et al.*, 2016 [139]	Etoricoxib	Double-blind, randomized, controlled study	64 female patients aged 18-75 with fibromyalgia syndrome	Adding 90 mg of etoricoxib (n = 32) or placebo (n = 32) daily to pre-existing antidepressants treatment combining placebo or pregabalin for 6 weeks	HDRS-17	There was no significant difference in HDRS-17 scores between combined treatment of etoricoxib and placebo
	Alamdarsaravi *et al.*, 2017 [135]	Celecoxib	Double-blind, randomized, placebo-controlled parallel-group, trial	40 patients aged 18-65 years with colorectal cancer over 100 days, receiving chemotherapy, with mild to moderate depression (HDRS-17 score 8-18)	Receiving standard colorectal cancer treatment, ingesting 200 mg celecoxib capsules (n = 20) or matched placebo (n = 20) twice daily for 6 weeks	HDRS-17	Celecoxib treatment resulted in significantly greater improvements than placebo in HDRS-17 scores by week 2 and remained progressively higher until week 6 post-intervention
	Berk *et al.*, 2020a [137]	Aspirin	Double-blind, randomized, placebo-controlled trial	19114 relatively healthy older adults aged ≥ 70 years	Daily usage of 100 mg of enteric-coated aspirin (n = 9525) or enteric-coated placebo (n = 9589) for 7 years	CES-D	Low-dose aspirin did not decrease the proportions of CES-D-10 scores ≥ 8 compared to placebo within the 7-year follow-up
	Berk *et al*., 2020b [138]	Rosuvastatin, Aspirin	Triple-blinded, randomized, parallel-group, controlled trial	138 young people aged 15-25 years, with moderate to severe MDD (MADRS score ≥ 20)	Ingesting 10 mg/day rosuvastatin (n = 42), 100 mg/day aspirin (n = 39), or placebo (n = 37) tablets for 12 weeks	MADRS	There were no significant differences in improvement of depression between rosuvastatin and placebo, or between aspirin and placebo. In patients aged ≤ 18 years, MADRS scores reduced more in rosuvastatin group at week 8, and in aspirin group at weeks 4 and 8, compared with placebo group. Statins were superior to aspirin on the MADRS at week 12
	Baune *et al.*, 2021 [136]	Celecoxib	Double-blind, randomized, placebo-controlled parallel-group trial	119 patients with MDD (MADRS score ≥ 20)	Vortioxetine supplemented with 400 mg/day celecoxib (n = 59) or placebo (n = 60) for 6 weeks	MADRS	Adjunctive celecoxib with vortioxetine for 6 weeks did not show better effects on depressive, cognitive and functioning symptoms, in depressive patients that mostly comprised treatment resistant individuals
Antibiotics	Emadi-Kouchak *et al.*, 2016 [147]	Minocycline	Double-blind, randomized, placebo-controlled, parallel-group study	46 HIV-infected patients with mild-to-moderate depression (HDRS-17 score ≤ 18)	Ingesting one tablet minocycline (100 mg/tablet) twice daily (n = 23) or matched placebo (n = 23) for 6 weeks added to highly active antiretroviral therapy (HAART)	HDRS-17	From baseline to week 3 and week 6, treatment with minocycline had significantly greater improvements in HDRS-17 total scores and more partial responders than that of placebo group
	Dean *et al.*, 2017 [148]	Minocycline	Double-blind, randomized, placebo-controlled trial	71 adult patients with MDD (MADRS score ≥ 25)	Ingesting one capsule minocycline (100 mg) twice daily with food (n = 36) or matched placebo (n = 35) for 12 weeks	MADRS	There was no significant difference in MADRS scores between adjunctive minocycline and placebo
	Husain *et al.*, 2017 [149]	Minocycline	Multi-site, double-blind, placebo-controlled, pilot trial	21 patients aged 18-65 years with MDE, and failed to respond to at least two antidepressant treatments	Minocycline (n = 21) or matched placebo (n = 20) added to treatment as usual for 12 weeks, started at a dose of 100 mg once daily and increased to 200 mg after 2 weeks	HDRS-17	Minocycline 200 mg/day added to treatment as usual for 12 weeks resulted in more significant improvement in depressive symptoms than placebo in patients with treatment-resistant MDE
Antibiotics	Abdallah *et al.*, 2020a [157]	Rapamycin	Double-blind, placebo-controlled, cross-over design study	20 patients aged 21-65 years with MDE (MADRS score ≥ 18)	Single pretreatment with oral 6 mg rapamycin or placebo 2 h prior to the intravenous injection of 0.5 mg/kg ketamine within 2 infusions, followed for 2 weeks (Who received rapamycin on Infusion 1 would receive placebo on Infusion 2, and vice-versa)	MADRS	Pretreatment with rapamycin did not alter the acute antidepressant effect of ketamine at 24 h post-treatment, but increased the response and remission rates at weeks 2
	Attwells *et al.*, 2021 [151]	Minocycline	Double-blind, randomized, placebo-controlled trial	21 participants with treatment-resistant MDE secondary to MDD	Minocycline (50 mg/day on week 1, 50 mg bid on week 2, and 100 mg bid on week 3-8) (n = 12) or placebo (n = 9) for 8 weeks, ^[18F]^FEPPA PET scans pre and post treatment	HDRS-17	Oral minocycline (up to 100 mg bid) had no significant effect on reduction of HDRS-17 scores and TSPO V_T_ within the PFC, ACC, insula and other assayed brain regions compared with placebo
	Nettis *et al.*, 2021 [150]	Minocycline	Double-blind randomized clinical trial	39 treatment-resistant depression (HDRS-17 score ≥ 14) patients with serum CRP ≥ 1 mg/L	Adjunctive minocycline (200 mg/day) (n = 18) or placebo (n = 21) added to ongoing antidepressant for 4 weeks	HDRS-17	Changes in HDRS-17 scores did not differ between study arms. Patients with baseline hsCRP levels ≥ 3 mg/L had the best reduction in HDRS-17 scores and the highest response rate
Antidiabetic drugs	Sepanjnia *et al.*, 2012 [160]	Pioglitazone	Double-blind, randomized, placebo-controlled, parallel-group study	40 patients aged 18-50 years with MDD (HDRS-17 score ≥ 22), with a score ≥ 2 on item 1	Adjunctive pioglitazone 15 mg (n = 20) or matched placebo (n = 20) every 12h with citalopram (20 mg/day for the first week, followed by 30 mg/day for subsequent 5 weeks) for 6 weeks	HDRS-17	Pioglitazone-treated patients had significantly lower HDRS-17 scores, as well as greater response and remission rates compared to the placebo group
	Lin *et al.*, 2015 [161]	Pioglitazone	Double blind, randomized, parallel- controlled trial	37 medically stable patients aged 23-71 years with non-psychotic, non-remitted depression	Treatment with 30 mg/day of pioglitazone (n = 19) or matched placebo (n = 18) pills for 12 weeks	HDRS-21	There was no statistically significant difference in mean reduction of HDRS-21 scores between groups. Improvement in depression was associated with improvement in glucose metabolism only in patients with insulin resistance
	Rasgon *et al.*, 2016 [162]	Pioglitazone	Double-blind randomized placebo-controlled trial	42 patients aged 23-71 with non-remitted depression who received at least 8 weeks stable treatment as usual	Adjunctive 30 mg pioglitazone (n = 22) daily or matched placebo (n = 20) pills added to treatment as usual for 12 weeks	HDRS-21	Subjects with longer telomeres exhibited greater declines in depression severity in the adjunctive pioglitazone treatment group, but not in the placebo group
	Abdallah *et al.*, 2020b [164]	Metformin	Double-blind placebo-controlled study	80 outpatients aged 23-57 years with MDD and HDRS-17 score > 18, with item 1 (depressed mood) score ≥ 2	Adjunctive metformin (1000 mg) (n = 40) or placebo (n = 40) tablet added to fluoxetine 20 mg once daily for 12 weeks	HDRS-17	After 4, 8 and 12 weeks of treatment, the improvement in HDRS-17 total scores was significantly higher in the metformin group than in the placebo group
Lipid regulating agents	Santanello *et al.*, 1997 [168]	Lovastatin	Double-blind randomized, clinical trial	431 participants aged ≥ 65 years with low density lipoprotein levels of 159-221 mg/dL	Ingesting diet plus placebo (n = 142), diet plus 20 mg/day of lovastatin (n = 144), or diet plus 40 mg/day of lovastatin (n = 145) for 6 months	CES-D	There were no statistically significant differences found in mean change of CES-D scores from baseline between treatment groups
	Stewart *et al.*, 2000 [170]	Pravastatin	Randomized placebo-controlled trial	1130 patients aged 31-74 years, with fasting serum cholesterol level of 4.0-7.0 mmol/L (155-271 mg/dL)	Treatment with pravastatin sodium 40 mg/day (n = 559) or matched placebo (n = 571) for at least 4 years	GHQ	During follow-up, there was no statistically significant difference in GHQ scores between treatment groups
	Carlsson *et al.*, 2002 [171]	Pravastatin, tocopherol	Double-blind, randomized, placebo-controlled, crossover study	41 community-dwelling participants aged ≥ 70 years with low-density lipoprotein-cholesterol (LDL-C) ≥ 3.62 mmol/L (140 mg/dL)	Receiving pravastatin for 6 months then pravastatin plus tocopherol for another 6 months (n = 20), or tocopherol for 6 months then pravastatin plus tocopherol for another 6 months (n = 21). Dosages were pravastatin 20 mg/day and tocopherol 400 IU/day	GDS	After 6 and 12 months, there were no significant changes in depression in the pravastatin, tocopherol, or combination treatment groups
Lipid regulating agents	Ghanizadeh and Hedayati, 2013 [169]	Lovastatin	Double blind, randomized, placebo-controlled trial	61 patients with MDD (HDRS-17 score ≥ 17)	Ingesting fluoxetine (up to 40 mg/day) plus 30 mg/day lovastatin (n = 34) or fluoxetine (up to 40 mg/day) plus placebo (n = 34) for 6 weeks	HDRS-17	Adjunctive treatment with lovastatin added to fluoxetine improved MDD symptoms more than placebo plus fluoxetine
	Haghighi *et al.*, 2014 [174]	Atorvastatin	Double-blind, randomized, placebo-controlled trial	60 inpatients aged 18-50 suffering from MDD (HDRS-21 score ≥ 25)	Wash-out for 2 weeks prior to treatment with 40 mg citalopram daily for 1 week, then treated with 20 mg/day atorvastatin (n = 30) or matched placebo (n = 30) added to continuous citalopram treatment for 12 weeks	HDRS-21	HDRS-21 scores significantly decreased over time. Compared to the placebo group, adjunctive treatment with atorvastatin had greater and faster improvement in depressive symptoms than placebo
	Abbasi *et al.*, 2015 [175]	Simvastatin, atorvastatin	Double-blind, randomized, placebo-controlled, parallel-group study	46 patients aged 18-50 years with a history of coronary artery bypass graft (CABG) in the last 6 months and MDD (HDRS-17 score ≤ 19)	Ingesting one tablet of simvastatin (20 mg/tablet) once daily (n = 23) or one atorvastatin tablet (20 mg/tablet) (n = 23) in the same manner for 6 weeks	HDRS-17	HDRS-17 scores decreased over time in both groups. Treatment with simvastatin for 6 weeks showed superior antidepressant effects compared to atorvastatin in patients undergoing CABG with mild to moderate depression
	Gougol *et al.*, 2015 [172]	Simvastatin	Double-blind placebo-controlled trial	44 patients aged 20-70 years with MDD (HDRS score ≥ 22)	Ingesting 20 mg/day simvastatin (n = 22) or placebo (n = 22) for 6 weeks added to fluoxetine (20 mg/day for the first two weeks followed by 40 mg/day for the subsequent four weeks)	HDRS-17	Adjunctive simvastatin-treated depressive patients experienced significantly more reductions and early improvement in HDRS-17 scores, and greater response rates compared to the placebo group
	Chan *et al.*, 2017 [173]	Simvastatin	Double-blind, parallel-group, randomized, placebo-controlled trial	140 patients aged 18-65 years with secondary progressive multiple sclerosis	Simvastatin 80 mg/day (two 40 mg tablets) (n = 70) or placebo (n = 20) (one tablet per day for the first month, while two per day from then on) for 24 months	HDRS	When combining all the patients, HDRS total scores increased significantly after the 24-month treatment, but there was no statistical difference between treatment groups
Glucocorticoids and endogenous substances	Arana *et al.*, 1995 [176]	Dexamethasone	Double-blind randomized, placebo-controlled trial	37 outpatients aged 18-70 years with MDD (HDRS-21 score > 20)	Treatment with 4 mg/day of oral dexamethasone (n = 19) or placebo (n = 18) for 4 days, follow-up for 14 days after the first dose of medication	HDRS-21	There were no statistically significant differences between groups in the HDRS scores, but the response rate in the dexamethasone group was greater than the placebo group
	DeBattista *et al.*, 2000 [177]	Hydrocortisone, corticotropin releasing hormone (CRH)	Double-blind, placebo-controlled study	21 patients with nonpsychotic major depression	Intravenously injection with 1 µg/kg of ovine CRH (n = 6), 15 mg of hydrocortisone (n = 6), or placebo saline (n = 10) for 2 days	HDRS-21	Patients treated with hydrocortisone demonstrated a significantly greater reduction in HDRS-21 scores than those given ovine CRH or placebo
	Ghazizadeh-Hashemi *et al.*, 2018 [179]	Palmitoylethanolamide	Double-blind, randomized, placebo-controlled trial	54 MDD patients aged 18-50 years (HDRS-17 score ≥ 19), with a score ≥ 2 on item 1 of HDRS	Adjunctive 600 mg PEA twice daily (n = 27) or placebo (n = 27) added to 40 mg/day citalopram for 6 weeks	HDRS-17	Add-on treatment with PEA showed greater improvement in depressive symptoms compared to placebo throughout the trial period

**Abbreviations:** QIDS-SR16, 16-item Quick Inventory of Depressive Symptomology - Self-Report; HDRS, Hamilton Depression Rating Scale; DASS-21, 21-item Depression, Anxiety, Stress Scale; SF-36, 36-item short form health survey questionnaire; ACC, Anterior cingulate cortex; BASDAI, Bath AS Disease activity index; BDI, Beck Depression Inventory; CES-D, Center for Epidemiological Studies-Depression scale; DHA, Docosahexaenoic acid; EPA, Eicosapentaenoic acid; ^[18F]^FEPPA, F 18-labeled N-(2-(2-fluoroethoxy)benzyl)-N-(4-phenoxypyridin-3-yl)acetamide); GHQ, General health questionnaire; GDS, Geriatric Depression Scale; HADS, Hospital Anxiety and Depression Scale; HIV, Human Immunodeficiency Virus; MDD, Major depressive disorders; MDE, Major depressive episode; MADRS, Montgomery-Asberg Depression Rating Scale; MCD, Multicentric Castleman’s disease; PEA, Palmitoylethanolamide; PHQ-9, Patient Health Questionnaire-9; PTX, Pentoxifylline; PUFA, Polyunsaturated fatty acid; POMS, Profile of Mood States; PET, Positron emission tomography; PFC, Prefrontal cortex; RA, Rheumatoid arthritis; TSPO, Translocator protein; ZDS, Zung Self-rating Depression Scale.

## LIST OF ABBREVIATIONS

5-HT: 5-hydroxytryptamine
ACC: Anterior Cingulate Cortex
AMPAR: α-amino-3-hydroxy-5-methyl-4-isoxazolepropionic acid glutamate receptor
AS: Ankylosing Spondylitis
BBB: Blood-brain Barrier
BDI: Beck Depression Inventory
BDNF: Brain-derived Neurotrophic Factor
CBT: Cognitive Behavioral Therapy
CES-D: Center for Epidemiological Studies Depression scale
CHM: Chinese Herbal Medicine
CNS: Central Nervous System
COMT: Catechol-O-methyltransferase
COX-2: Cyclooxygenase-2
CRH: Corticotropin-releasing Hormone
DALYs: Disease Burden Using Disability-adjusted Life Years
DAMPs: Danger-associated Molecular Patterns
DHA: Docosahexaenoic Acid
DSM: Diagnostic and Statistical Manual of Mental Disorders
ECT: Electroconvulsive Therapy
EPA: Eicosapentaenoic Acid
FDA: Food and Drug Administration
FDG: 18-Fluorodeoxyglucose
GDS: Geriatric Depression Scale
GHQ: General Health Questionnaire
GR: Glucocorticoid Receptor
GZA: Glycyrrhizic Acid
HADS: Hospital Anxiety and Depression scale
HDRS: Hamilton Scale for Depression
HMG-CoA: 3-hydroxy-3-methylglutaryl-coenzyme A
HMGB1: High Mobility Group Box 1 Protein
HPA: Hypothalamic-pituitary-adrenal
HRQOL: Health-related Quality of Life
hs-CRP: High Sensitive C-reaction Protein
HSP: Heat Shock Proteins
IFN-α: Interferon-α
IgG1: Immunoglobulin 1
IL-10: Interleukin-10
IL-1β: Interleukin-1β
IL-4: Interleukin-4
IL-6: Interleukin-6
LDL: Low Density Lipoprotein
LTL: Leukocyte Telomere Length
MADRS: Montgomery-asberg Depression Rating Scale
MAOIs: Monoamine Oxidase Inhibitors
MAPK: Mitogen-activated Protein Kinase
MBCT: Mindfulness-based Cognitive Therapy
MBI: Mindfulness-based Intervention
MBSR: mindfuLness-based Stress Reduction
MDD: Major Depressive Disorder
MINI: Mini-international Neuropsychiatric Interview
MLR: Monocyte-to-lymphocyte Ratio
mTORC1: Mechanistic Target of Rapamycin Complex 1
NaSSAs: Noradrenergic and Specific Serotonergic Antidepressants
NDRIs: Norepinephrine-dopamine Reuptake Inhibitors
NLR: Neutrophil-to-lymphocyte Ratio
NMDAR: N-methyl-D-aspartate Receptor
NSAIDs: Nonsteroidal Anti-inflammatory Drugs
OGTT: Oral Glucose Tolerance Test
PAMPs: Pathogen-associated Molecular Patterns
PEA: Palmitoylethanolamide
PET: Positron Emission Tomography
PFC: Prefrontal Cortex
PGs: Prostaglandins
PLR: Platelet-to-lymphocyte Ratio
PPAR-γ: Peroxisome Proliferator Activator Gamma Receptor
PROs: Patient-reported Outcomes
PRRs: Pattern Recognition Receptors
PUFA: Polyunsaturated Fatty Acid
QIDS-SR16: Quick Inventory of Depressive Symptomology - Self-Report
RA: Rheumatoid Arthritis
rTMS: Repetitive Transcranial Magnetic Stimulation
SFDA: State Food and Drug Administration
SGLT2i: Sodium-glucose Cotransporter Type 2 Inhibitor
sICAM1: Soluble Intracellular Adhesion Molecule-1
SNRIs: Serotonin Noradrenaline Reuptake Inhibitors
SSRIs: Selective Serotonin Reuptake Inhibitors
TCAs: Tricyclic Antidepressants
TCM: Traditional Chinese Medicine
tDCS: Transcranial Direct Current Stimulation
TeCAs: Tetracyclic Antidepressants
Th17: T helper cell 17
TNF-α: Tumor Necrosis Factor-α
TRD: Treatment-resistant Depression
TSPO: Translocator Protein
TZDs: Thiazolidinediones
ZDS: Zung Self-Rating Depression Scale.
